# Tuning charge density of chimeric antigen receptor optimizes tonic signaling and CAR-T cell fitness

**DOI:** 10.1038/s41422-023-00789-0

**Published:** 2023-03-08

**Authors:** Jian Chen, Shizhen Qiu, Wentao Li, Kun Wang, Yu Zhang, Han Yang, Baichuan Liu, Guangfei Li, Li Li, Min Chen, Junjie Lan, Jiahua Niu, Peijie He, Lei Cheng, Gaofeng Fan, Xin Liu, Xianmin Song, Chenqi Xu, Haitao Wu, Haopeng Wang

**Affiliations:** 1 School of Life Science and Technology, ShanghaiTech University, Shanghai, China; 2 ENT institute and Department of Otorhinolaryngology, Eye & ENT Hospital, Fudan University, Shanghai, China; 3 State Key Laboratory of Molecular Biology, Shanghai Institute of Biochemistry and Cell Biology, Center for Excellence in Molecular Cell Science, Chinese Academy of Sciences, University of Chinese Academy of Sciences, Shanghai, China; 4 Xiamen Shuangshi Middle School of Fujian, Xiamen, Fujian China; 5 Department of Hematology, Shanghai General Hospital, Shanghai Jiaotong University School of Medicine, Shanghai, China; 6 School of Life Science, Hangzhou Institute for Advanced Study, University of Chinese Academy of Sciences, Hangzhou, Zhejiang China; 7 Shanghai Clinical Research and Trial Center, Shanghai, China; 8 Present Address: CeMM, Research Center for Molecular Medicine of the Austrian Academy of Sciences, Vienna, Austria

**Keywords:** Tumour immunology, Cancer immunotherapy

## Abstract

Tonic signaling of chimeric antigen receptor (CAR), i.e., the spontaneous CAR activation in the absence of tumor antigen stimulation, is considered to be a pivotal event controlling CAR-T efficacy. However, the molecular mechanism underlying the spontaneous CAR signals remains elusive. Here, we unveil that positively charged patches (PCPs) on the surface of the CAR antigen-binding domain mediate CAR clustering and result in CAR tonic signaling. For CARs with high tonic signaling (e.g., GD2.CAR and CSPG4.CAR), reducing PCPs on CARs or boosting ionic strength in the culture medium during ex vivo CAR-T cell expansion minimizes spontaneous CAR activation and alleviates CAR-T cell exhaustion. In contrast, introducing PCPs into the CAR with weak tonic signaling, such as CD19.CAR, results in improved in vivo persistence and superior antitumor function. These results demonstrate that CAR tonic signaling is induced and maintained by PCP-mediated CAR clustering. Notably, the mutations we generated to alter the PCPs maintain the antigen-binding affinity and specificity of the CAR. Therefore, our findings suggest that the rational tuning of PCPs to optimize tonic signaling and in vivo fitness of CAR-T cells is a promising design strategy for the next-generation CAR.

## Introduction

Chimeric antigen receptor (CAR) is a synthetic antigen receptor targeting T lymphocytes to specifically attack tumor cells. CAR is composed of an extracellular domain for tumor antigen recognition, in which the variable regions of the heavy and light chains of an antibody are connected by a flexible linker to form a single-chain variable fragment (scFv), and an intracellular domain for triggering T cell activation, which contains the signaling domains of T cell receptor (TCR) and costimulatory receptors. To date, CAR-T cell therapies have shown unprecedented efficacy in B cell malignancies but poor efficacy against solid tumors, mainly due to the limited in vivo persistency of CAR-T cells and impaired T cell function while treating solid tumors.^1^

TCR constantly interacts with self-antigens in vivo without foreign antigens, generating low but constitutive signals known as tonic signaling.^2^ TCR tonic signaling benefits several cellular processes of T cells, including T cell homeostasis, survival, and differentiation.^3^ Similarly, CAR-T cells exhibit cell-autonomous tonic signaling, characterized by spontaneous but weak cell activation and low-level release of pro-inflammatory cytokines in the absence of tumor antigen stimulation. Unlike TCR, the effects of CAR tonic signaling are context-dependent. Strong CAR tonic signaling can cause rapid T cell exhaustion, impairing antitumor function.^4,5^ Alternatively, inefficient CAR tonic signaling can also lead to limited efficacy. Increased tonic signaling improves CAR-T therapy efficacy in the setting of CD22.BBζ CAR, which exhibits weak CAR tonic signaling.^6^ A modified CD22.CAR design with a higher level of tonic signaling than the original design displays better immune synapse formation, enhanced activation of pro-inflammatory genes, and superior antitumor function, all of which contribute to a better clinical outcome. These findings imply that the strength of CAR tonic signaling should be fine-tuned for optimal antitumor function and improved clinical efficacy. Several approaches, including replacing the costimulatory module, optimizing the stability of the framework of scFv, and shortening the linker connecting the variable regions of the heavy and light chains, have been applied to adjust CAR tonic signaling.^4,6,7^ However, how CAR tonic signaling is initiated and how its strength is regulated remain elusive. Addressing these basic questions might promote the rational improvement of current CAR-T therapies.

Ligand-independent tonic signaling is a common feature of many antigen receptors and is critical for lymphocyte development.^8^ The pre-B cell receptor (pre-BCR), composed of a heavy chain and an invariant surrogate light chain, is able to generate tonic signals in the absence of ligands.^9^ Self-aggregation of the pre-BCR induced by electrostatic interaction between themselves has been proposed to explain the generation of this tonic signal. Mutation of seven evolutionarily conserved, positively charged arginine residues in the N-terminal portion of λ5 chain, a component of the surrogated light chain, abolished the pre-BCR tonic signaling.^10^ Interestingly, in the λ5-deficient patient, some pre-BCRs containing the unusual heavy chains that are significantly enriched for the positively charged residues in the CDR3 region could drive early B cell development in the absence of the surrogate light chain.^11^ Like pre-BCR, pre-TCR, composed of the pre-TCR α-chain (pTα) and TCR β-chain, can also deliver a tonic signal autonomously, which is essential for thymocyte development. The pre-TCR tonic signaling also relies on the autonomous oligomerization of pre-TCR mediated by some positively charged residues that are evolutionally conserved in pTα.^12,13^ These facts collectively suggest that the electrostatic interaction of antigen receptors often plays an important role in triggering autonomous signals. However, whether the electrostatic interaction is involved in initiating and regulating the tonic signaling of CAR, a synthetic antigen receptor, is not known.

Here we first characterized the surface charge distribution of ten different CARs currently being studied in clinical settings. We discovered a significant correlation between the strength of tonic signaling and the enrichment of positively charged residues on the surface of CAR antigen-binding domains. Furthermore, modulating the tonic signaling by tuning PCPs improves the fitness and antitumor function of CAR-T cells. Our study provides a straightforward approach for optimizing CAR tonic signaling, without compromising antigen-induced CAR signaling.

## Results

### CAR-T exhaustion induced by high tonic signaling of commonly used CARs

**Fig. 1:**
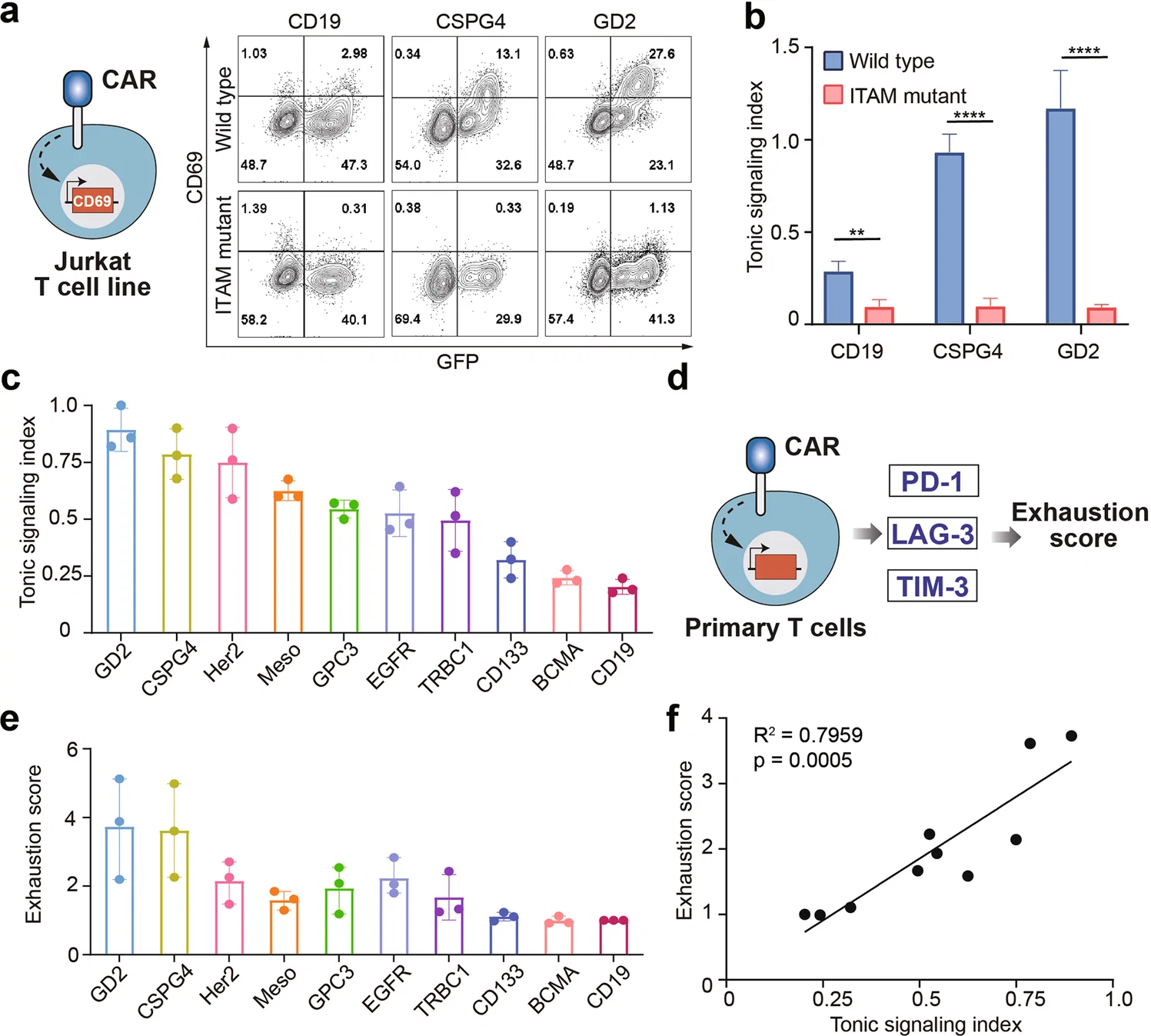
CAR-T exhaustion induced by high tonic signaling of many commonly used CARs. **a** FACS analysis of CD69 expression level of Jurkat T cells expressing wild type and ITAM mutant CD19, CSPG4, and GD2.CARs. **b** CD69/GFP, defined as the tonic signaling index, of the three CARs shown in **a**. **c** The tonic signaling index of ten commonly used CARs. **d** The average expression of LAG3, TIM3 and PD-1 on CAR-T cells used to define the exhaustion score. **e** The exhaustion score of the CAR shown in **c**. **f** Correlation between the exhaustion score and the tonic signaling index in these CAR-T cells assessed by the Pearson method. Data are presented as means ± SEM. All comparisons were determined using Student’s *t*-tests; **P < 0.01; ****P < 0.0001.

To set up a robust assay to quantitate the strength of CAR tonic signaling, we expressed the CAR construct containing a GFP as a reporter in the Jurkat cells, a human T leukemia cell line (Supplementary information, Fig. S1a). As a readout of CAR signaling-induced activation, we measured the up-regulation of CD69 (Fig. 1a), a well-defined early marker of T cell activation previously used to indicate the strength of pre-TCR and pre-BCR tonic signaling.^11,12^ We further defined a “tonic signaling index” for each sample by calculating the expression level of CD69 normalized by GFP expression, an indicator of CAR expression. GD2.CAR and CSPG4.CAR are two known CARs with high tonic signaling, while CD19.CAR is reported to have low tonic signaling.^4,7^ As expected, the expression of GD2.CAR or CSPG4.CAR induced much higher CD69 expression than CD19.CAR in the absence of the tumor antigen stimulation (Fig. 1a). Having comparable expression levels as wild type (WT)-CARs (Supplementary information, Fig. S1b), immunoreceptor tyrosine-based activation motif (ITAM) deficient-CARs failed to induce CD69 expression, indicating that CD69 upregulation in the current assay indeed resulted from ITAM-mediated tonic signaling (Fig. 1a, b).^14^ In addition, we observed that the transduction efficiency minimally affected the tonic signaling index (Supplementary information, Fig. S1c).

Next, we selected 10 commonly used CARs targeting GD2, Her2, CSPG4, EGFR, Mesothelin, GPC3, TRBC1, CD133, BCMA, and CD19, respectively, and calculated their tonic signaling index. All CAR constructs used identical sequence containing the CD8 hinge-transmembrane region and the CD28 costimulatory domain followed by the CD3ζ intracellular domain, except for GD2.CAR and CSPG4.CAR, where the CD8 hinge-transmembrane region was replaced with the IgG1 CH2CH3 region and CD28 transmembrane region to achieve stable surface expression (Supplementary information, Fig. S1a).^15^ In the case of TRBC1.CAR, it is known that each T cell chooses to express a TCRβ-chain constant region encoded by either TRBC1 or TRBC2, in a mutually exclusive manner.^16^ Since Jurkat T cell line is TRBC1^+^, we used a TCR-knockout Jurkat cell line to measure the tonic signaling index of the TRBC1.CAR (Supplementary information, Fig. S1d). Consistent with the previous report,^17^ we also confirmed that there is little or no surface expression of CD133 in either Jurkat T cells or primary T cells (Supplementary information, Fig. S1e).

We found that each CAR displayed variable tonic signaling strength: CD19.CAR with the lowest and GD2.CAR having the highest (Fig. 1c). Unlike the CD19.CAR, spontaneous exhaustion is present in most solid-tumor CARs and may contribute to the poor efficacy of CAR-T cells targeting solid tumors in current clinical trials.^4^ To explore the potential relationship between tonic signaling strength and CAR-T cell exhaustion, we generated the primary CAR-T cell using these 10 different CARs. In order to avoid self-antigen-induced TRBC1.CAR signaling, we sorted TRBC1^–^ primary T cells and expressed the TRBC1.CAR on these T cells (Supplementary information, Fig. S1d). Without antigen triggering, the exhaustion markers, including PD-1, Tim-3, and Lag-3, were upregulated in these 10 different CAR-T cells at variable degrees (Supplementary information, Fig. S1f–h). Meanwhile, as previously reported, some exhausted CAR-T cells (e.g., GD2.CAR-T cells) also lost cytokine production and showed impaired killing ability in vivo (Supplementary information, Fig. S1i–k). We calculated an “exhaustion score” for each type of CAR-T cell by taking the average expression of three exhaustion markers, including PD-1, Tim-3, and Lag-3, for direct comparison (Fig. 1d, e). We found a significant positive correlation between the exhaustion score and the tonic signaling index in these CAR-T cells (r^2^ = 0.7959, *P* < 0.001, Fig. 1f). These results demonstrate that the tonic signaling index faithfully reflects the strength of CAR tonic signaling and confirm that sustained antigen-independent tonic signaling induces the exhaustion of CAR-T cells.^4,18–20^

### PCPs on the antigen-binding domain determine CAR tonic signaling strengths

**Fig. 2:**
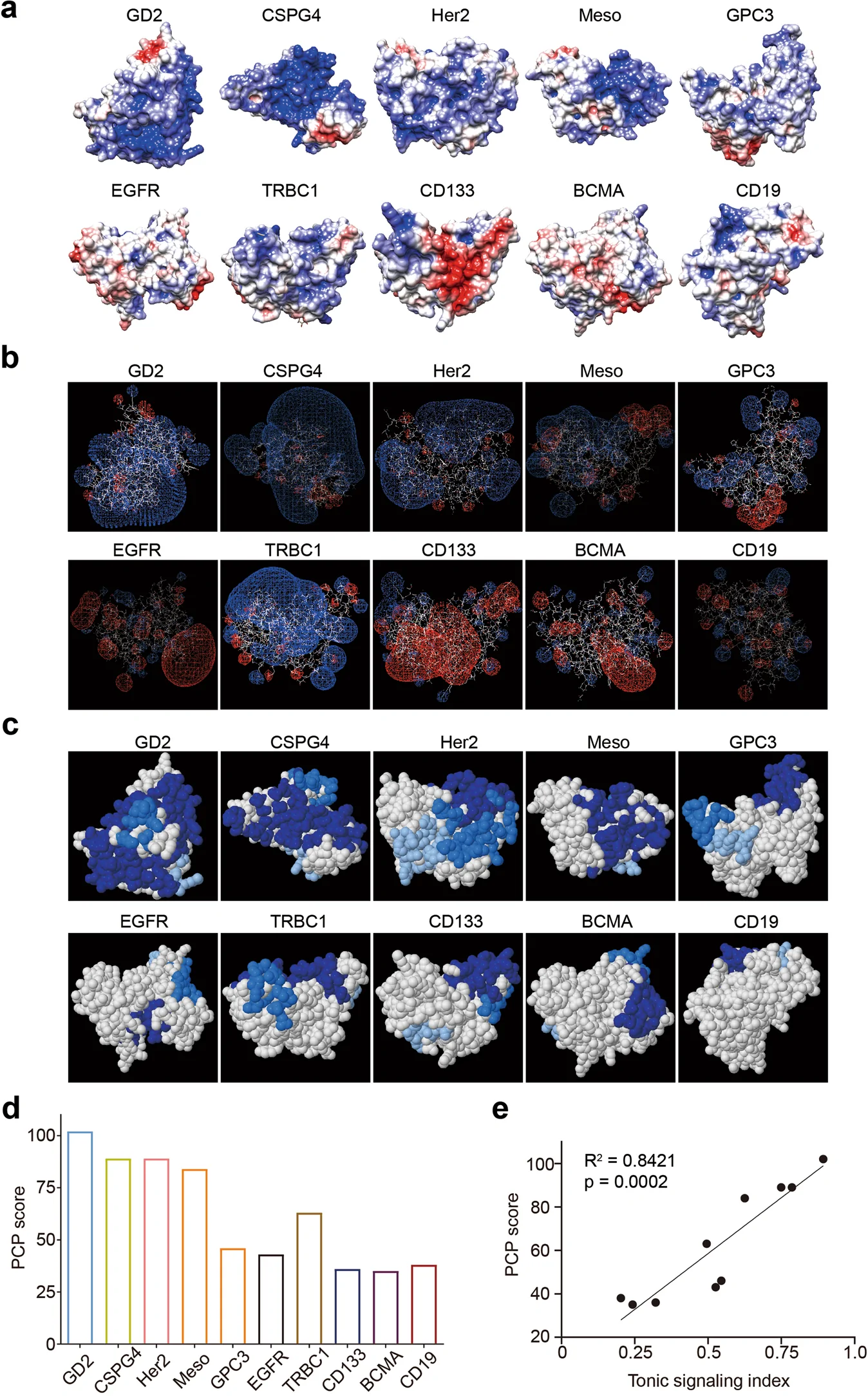
PCPs on the antigen-binding domain surface determine CAR tonic signaling strengths. **a** Electrostatics analysis of the ten CAR scFv constructs using APBS within UCSF Chimera. Blue, positively charged surface; red, negatively charged surface. **b** The electrostatic potential fields of the charged CAR scFv observed in the Swiss-PDBViewer software. **c** The top three largest patches containing continuous positive charged residues displayed by the BindUP web server tool. Dark blue, the first-largest PCP; medium blue, the second-largest PCP; light blue, the third-largest PCP. **d** The PCP score, which represents the sum of all residues within the top three largest PCPs, of the CAR scFvs shown in **c**. **e** Correlation between the PCP score and the tonic signaling index in these CAR-T cells, assessed by the Pearson method.

The 10 different CARs with nearly identical structures except for the antigen-binding domain, scFv, displayed distinct levels of tonic signaling strength, indicating that the CAR signaling strength might depend on the scFv region of CARs (Fig. 1c; Supplementary information, Fig. S1a). To exploit the potential biophysical properties of CAR scFvs driving CAR tonic signals, we first constructed three dimensional (3D) homology models for all CAR scFvs tested using the SWISS homology modeler (Supplementary information, Fig. S2a). As noted in the introduction, the presence of positively charged residues induces spontaneous oligomerization and activation of pre-BCRs and pre-TCRs.^10–12^ Therefore, we tested whether the electrostatic properties of CARs could contribute to CAR tonic signals. We calculated the surface electrostatic profiles of CAR scFvs using APBS and displayed the results using UCSF Chimera (Fig. 2a). There are two interesting observations. First, we observed more positively charged residues on CAR scFv surfaces with a high tonic signaling index as compared with those with a low one (Supplementary information, Fig. S2b). Correlation analysis revealed a weak but significant correlation between the net positive charge per amino acid and CAR tonic signaling index (r^2^ = 0.5057, *P* < 0.05, Supplementary information, Fig. S2c). Second, the positively charged residues intend to form patches on the surface of those CARs with high tonic signaling, which can induce a stronger electrostatic field around the molecule (Fig. 2b). To quantitatively measure the positively charged patches (PCPs) on each CAR surface, we calculated the PCP score, the total number of all residues within the top three largest patches containing continuous positively charged residues displayed by the BindUP web server tool (Fig. 2c, d). Impressively, a statistically significant linear relationship exists between the PCP score and CAR tonic signaling index (r^2^ = 0.8421, *P* = 0.0002), suggesting a potential role of PCPs in CAR tonic signaling (Fig. 2e).

It was also recently reported that CAR tonic signaling was likely caused by the intrinsic instability of the scFv that promotes CAR self-aggregation.^7^ Therefore, we also evaluated the molecular stabilities of the scFv domain of each CAR. Using the protein-sol server (https://protein-sol.manchester.ac.uk/), we predicted the protein stabilities of CAR scFvs in a physiological-chemical environment (0.15 ionic strength and pH 7.5, Supplementary information, Fig. S2d). Quantification of scFv stability relied on the energy given in Joules per amino acid, a normalization against protein size. As shown in Supplementary information, Fig. S2e, no significant correlations between CAR tonic signaling indexes and scFv stabilities were observed (*P* > 0.1, Supplementary information, Fig. S2e), indicating instability of scFV is not the major biophysical property that drives CAR tonic signaling. Together, these results suggest that the electrostatic interactions induced by PCPs on CARs may play a key role in regulating tonic signaling strength.

### Optimization of CAR tonic signaling and CAR-T fitness via adjusting ionic strength during ex vivo expansion

**Fig. 3:**
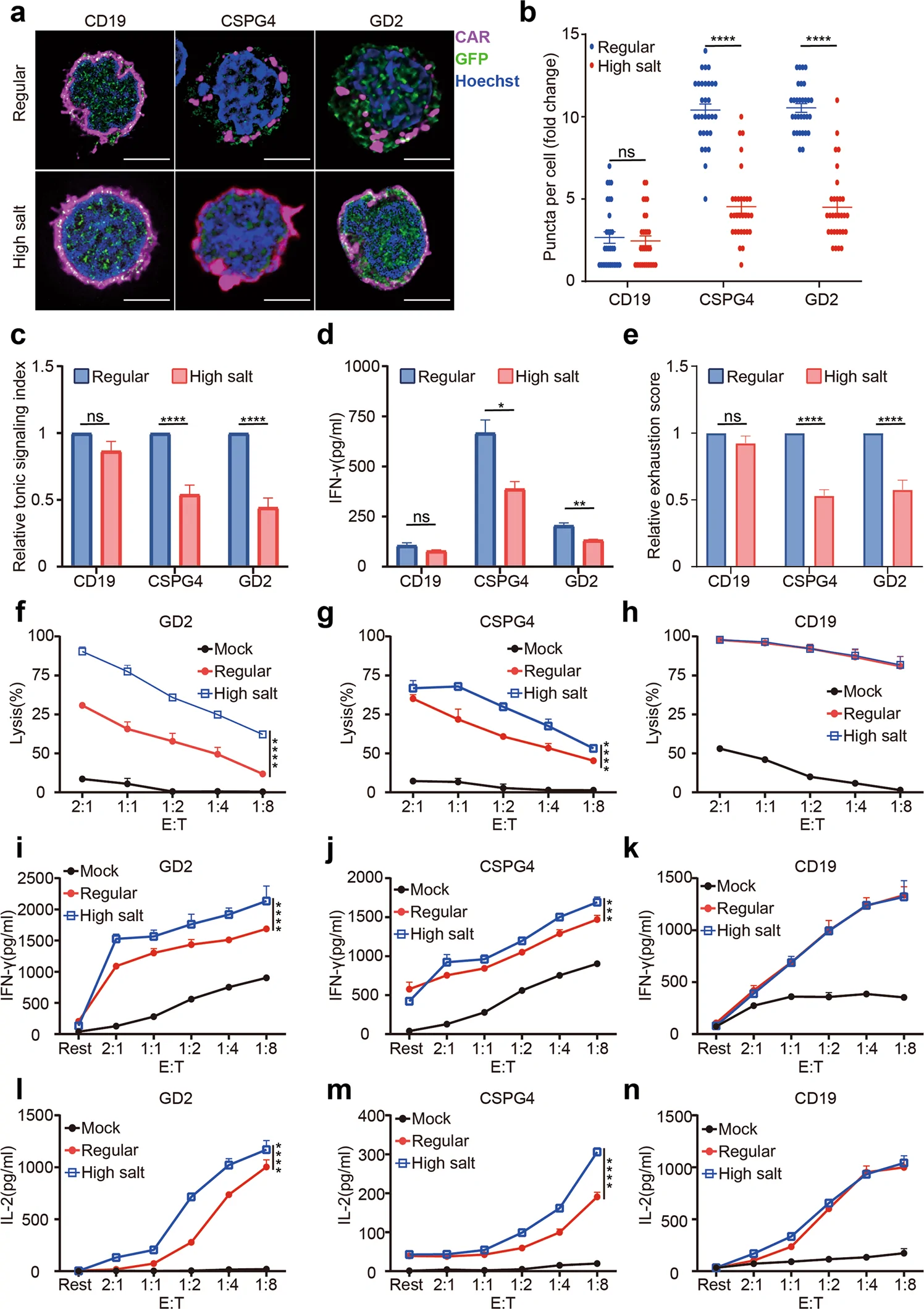
Adjusting ionic strength during ex vivo expansion to optimize CAR tonic signaling and CAR-T function. **a** Imaging analysis of CAR clustering of Jurkat T cells expressing CD19, CSPG4, or GD2.CARs cultured from regular or H.S. medium without antigen stimulation. Pink, CAR; green, CAR-IRES EGFP; blue, Hoechst. Scale bars, 5 μm. **b** Analysis of the number of the CAR puncta per cell shown in **a**. **c** Jurkat T cells expressing indicated CAR were cultured in either regular or H.S. medium and CAR tonic signaling indexes normalized by the index of sample grown in regular medium were shown. **d** IFN-γ secretion levels of CD19, CSGP4, and GD2.CAR-T cells cultured in regular or H.S. medium without antigen stimulation. **e** Primary T cells expressing indicated CAR were cultured in either regular or H.S. medium and CAR exhaustion scores normalized by the score of samples grown in regular medium were shown. **f**–**h** In vitro cytotoxicity assay of CAR-T cells expressing CD19, CSPG4, and GD2.CARs. Indicated CAR-T cells grown in regular or H.S. medium were harvested and co-incubated with their target tumor cells at the indicated E:T ratio overnight. Killing efficiency was analyzed by luciferase cytotoxicity assay. **i**–**n** Cytokine secretion assay of CAR-T cells after activation. Indicated CAR-T cells grown in regular or H.S. medium were harvested and co-incubated with their target tumor cells at the indicated E:T ratio. The levels of IL-2 and IFN-γ were determined by ELISA. Data are presented as means ± SEM. Comparisons were determined using unpaired Student’s *t*-tests (**b**–**e**) or two-way analysis of variance (**f**–**n**); *P < 0.05; **P < 0.01; ***P < 0.001; ****P < 0.0001; ns, not significant.

Previous studies demonstrated that the net charge of pre-TCR or BCR induces their oligomerization mediated by electrostatic interactions.^10–12^ To examine whether electrostatic interactions between CAR scFvs lead to CAR clustering, we detected the antigen-independent oligomerization of CARs with different charge properties by using microscopy. Expectedly, both the GD2.CAR and CSPG4.CAR, which contain predominantly positively charged patches on their scFv surfaces, were aggregated in punctate on the T cell membrane. In contrast, CD19.CARs with even distribution of positively electrostatic fields displayed a uniform distribution on the T cell surface (Fig. 3a, b). This process was not due to the secondary effect of CAR tonic signaling since similar punctate phenotypes were also observed in the T cells expressing ITAM-deficient GD2.CAR or CSPG4.CARs (Supplementary information, Fig. S3a).

The electrostatic property of CAR surface controlling CAR oligomerization and tonic signaling leads to the prediction that high CAR tonic signaling will be reduced when enhancing the ionic strength in the CAR-T culture medium, considering the fact that the effective charge on proteins in solution could decrease due to the electrostatic charge shielding.^21^ To test this prediction, we increased the concentration of sodium chloride (NaCl) in the ex vivo culture medium. As shown in Fig. 3a, b, both GD2. and CSPG4.CARs were significantly prevented from clustering after adding 50 mM NaCl into the medium. Interestingly, high-salt (H.S.) treatment did not affect CAR signal transduction upon tumor antigen engagement but significantly reduced the tonic signaling index (Fig. 3c; Supplementary information, Fig. S3b–e). Overall, these results were consistent with a model in which CAR tonic signaling results from antigen-independent electrostatic interactions between CAR scFv domains, leading to their self-association on the T cell surface.

Currently, an about two-week ex vivo expansion of CAR-T cells is normally required to generate enough engineered T cells with antitumor function preserved for re-infusion into patients.^22^ Primary T cells expressing high tonic signaling CAR often result in functional exhaustion, undergoing a progressive loss of cytokine production and cytotoxicity. We thought to test if adjusting ionic strength in the ex vivo culture medium could improve the function of highly tonic CAR-T cells. When grown in H.S. media, highly tonic CAR-T cells showed lower amounts of spontaneous cytokine production and lower exhaustion scores than when grown under normal conditions (Fig. 3d, e; Supplementary information, Fig. S3f–h). Primary T cells expressing GD2.CARs cultured in H.S. medium exhibited a superior killing ability and a stronger secretion of IL-2 and IFN-γ against GD2^+^ neuroblastoma cell line CHLA-255 in vitro compared to those cultured in a regular medium (Fig. 3f, i, l). Similar results were also observed in the CSPG4.CAR-T cells killing assay using CSPG4^+^ nasopharyngeal cancer cell line CNE-2 (Fig. 3g, j, m). Yet, the cytotoxicity activity and cytokine secretion ability of CD19.CAR-T cells cultured in the H.S. medium were barely affected (Fig. 3h, k, n). Hence, adjusting the ionic strength in the ex vivo culture medium represents a convenient and straightforward approach for producing functional T lymphocytes expressing highly tonic CARs against solid tumors.

### Tuning down PCPs on the CAR surface mitigates T cell exhaustion induced by high tonic signaling

To decipher the direct association between PCPs on the CAR surface and CAR tonic signaling, we attempted to mutate positively charged amino acids of CAR scFv to reduce electrostatic interactions between CARs. An scFv is segregated into complementarity determining regions (CDRs), which bind directly to antigens, and framework regions (FRs), which act as a scaffold for CDRs.^23^ Given that FRs usually constitute about 85% of the scFv sequences but do not contact antigen directly,^23^ we mutated several lysines of FRs to uncharged residues inside or near PCPs on GD2.CAR (Supplementary information, Fig. S4a). Compared to WT GD2.CAR, these GD2.CAR mutants with reduced net charge exhibited weaker positive electric fields and reduced PCP scores (Supplementary information, Fig. S4b, c). Nearly all the mutants displayed significantly diminished tonic signaling strength, among which mutant GD2^F^ with relatively low tonic signaling was chosen for the 2^nd^ round of optimization (Supplementary information, Fig. S4d).

**Fig. 4:**
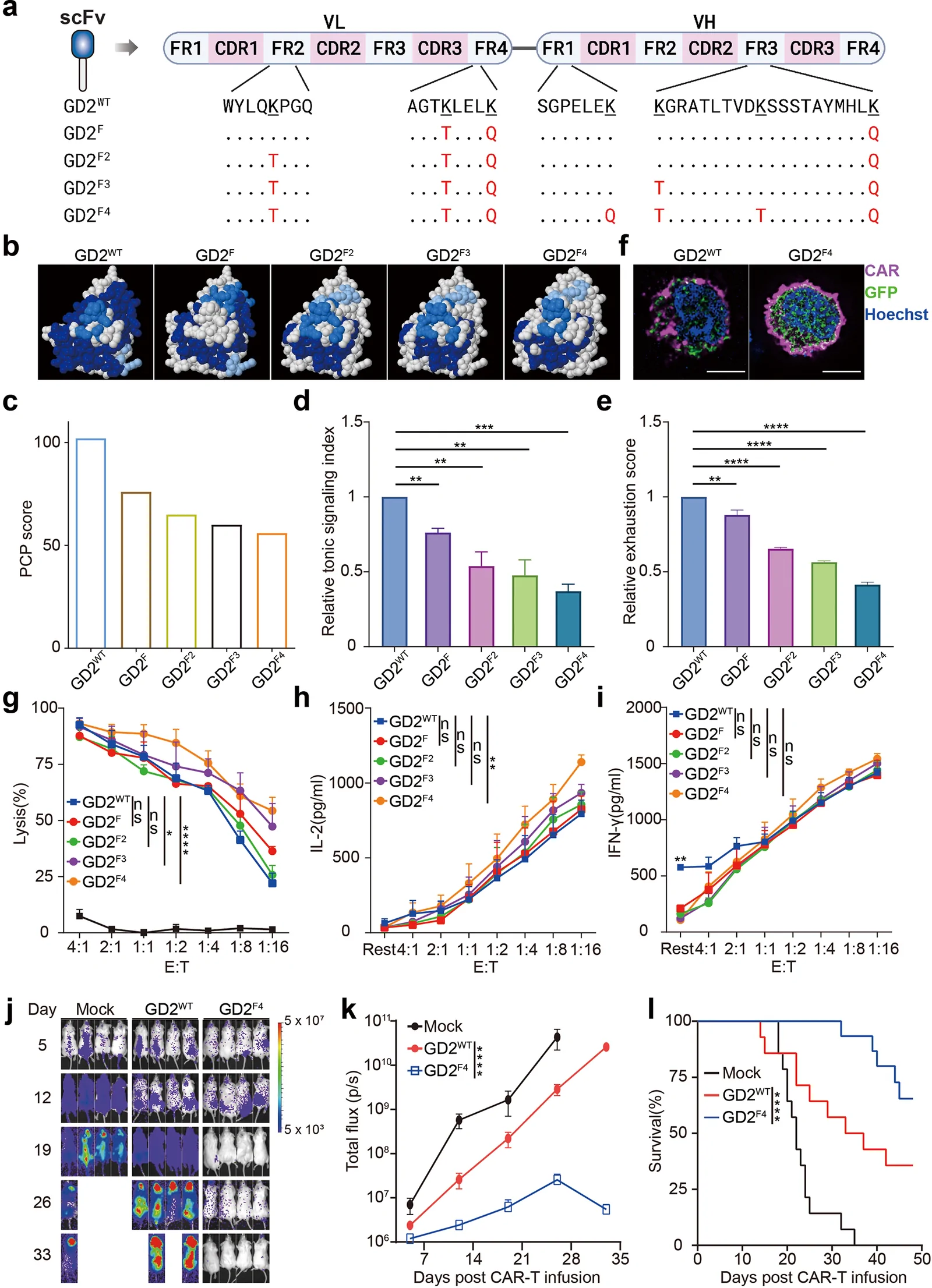
Reducing PCPs on the CAR surface mitigates T cell exhaustion induced by high CAR signaling. **a** The sequences of optimized GD2.CAR variants. **b** The top three largest PCPs on optimized GD2.CAR scFv surface displayed by the BindUP web server tool. Dark blue, the first-largest PCP; medium blue, the second-largest PCP; light blue, the third-largest PCP. **c** The calculation of PCP scores for the optimized GD2.CAR scFvs. **d** The calculation of relative tonic signaling indexes for the optimized GD2.CAR scFvs. **e** The calculation of relative exhaustion scores for the optimized GD2.CAR scFvs. **f** Imaging analysis of CAR clustering on GD2^WT^ and GD2^F4^.CAR-T cells. Pink, CAR; green, CAR-IRES EGFP; blue, Hoechst. Scale bars, 5 μm. **g** In vitro killing assay against GD2^+^ neuroblastoma cell line CHLA-255 of WT and mutated GD2.CAR-T cells. **h**, **i** Cytokine secretion assay of CAR-T cells after activation. WT and optimized GD2.CAR-T cells were co-incubated with their target tumor cells at the indicated E:T ratio. The levels of IL-2 and IFN-γ were determined by ELISA. **j** Representative bioluminescence images of tumor burden after GD2^WT^ and GD2^F4^.CAR-T infusion over time. **k** Quantifications of luciferase intensity, which reflets tumor burden, of each mouse shown in **j** using IVIS system. **l** Survival curves for the mice shown in **j**. Data are presented as means ± SEM; Comparisons were determined using unpaired Student’s *t*-tests (**d**, **e**), two-way analysis of variance (**g**–**i**, **k**), and survival analysis (**l**); *P < 0.05; **P < 0.01; ***P < 0.001; ****P < 0.0001; ns, not significant.

Four additional lysines in GD2^F^.CAR were targeted for further mutagenesis, which generates three new mutants named GD2^F2–4^ (Fig. 4a). Compared to the original GD2^F^, these new CAR mutants have less positively charged residues and form weaker electrostatic potential fields (Fig. 4b; Supplementary information, Fig. S4e, f). Interestingly, along with more lysines being mutated, these mutants exhibit decreased PCP scores, tonic signaling index and exhaustion scores in a stepwise manner (Fig. 4c–e; Supplementary information, Fig. S4g–i). Among these mutants, the GD2 ^F4^ mutant that has the lowest PCP score completely abolished spontaneous CAR clustering (Fig. 4f). Furthermore, we also observed that the percentage of TCF1^+^ progenitor exhausted cells (Tpex) was significantly enriched in Lag3^+^TOX^+^ exhausted GD2^F4^.CAR-Ts compared to GD2^WT^.CAR-Ts (Supplementary information, Fig. S4j). Consequently, GD2^F4^.CAR-T cells killed tumor cells more potently in vitro (Fig. 4g). Meanwhile, with a lower basal level of cytokine secretions, GD2^F4^.CAR-T cells could secret higher levels of IL-2 and IFN-γ after activation than WT CAR-T cells (Fig. 4h, i).

To explore the signaling basis that mediates the effects of modulating PCPs, we compared MAPK and mTOR activities downstream of the CAR tonic signal. Phosphorylation of ERK, a signature biochemical event of the MAPK pathway, and phosphorylation of two mTOR substrates (S6 and 4E-BP1), indicators of mTOR activity, were quantitated by intracellular fluorescence-activated cell sorting (FACS) staining (Supplementary information, Fig. S4k–n). We found that the activities of both the MAPK and mTOR pathways were enhanced in CAR-expressing T cells compared to mock T cells, suggesting that both pathways are downstream of CAR tonic signaling. Moreover, the MAPK activities were significantly inhibited in GD2^F4^.CAR-T cells compared to that in GD2^WT^.CAR-T cells, indicating a potential role for the MAPK pathway in tonic signaling-induced CAR-T exhaustion.

Finally, we tested the functional improvement of GD2^F4^.CAR-T cells in vivo, using a tumor model established previously.^5^ As shown in Fig. 4j–l, GD2^+^ tumor cells were efficiently cleared after GD2^F4^.CAR-T infusion, whereas GD2^WT^.CAR-T cells-transferred mice exhibited uncontrollable tumor progression and shortened survivals.

PCP reduction could also be achieved by CDR grafting. Take CSPG4.CAR that has the second-highest tonic signaling index as an example. We replaced its positively charged FR with less charged FRs (Supplementary information, Fig. S4o), which significantly inhibited CSPG4.CAR-T tonic signaling strength (Supplementary information, Fig. S4p–r). As a representative optimization, CSPG4^M5^.CAR-T cells had decreased autonomous CAR clustering (Supplementary information, Fig. S4s), and showed a superior anti-tumor efficacy both in vitro and in vivo (Supplementary information, Fig. S4t, u). Our findings indicated that PCP reduction is an efficient method to improve the CAR design with high tonic signaling.

### Tuning up PCPs on the surface of CARs with inefficient tonic signaling promotes CAR-T persistence

**Fig. 5:**
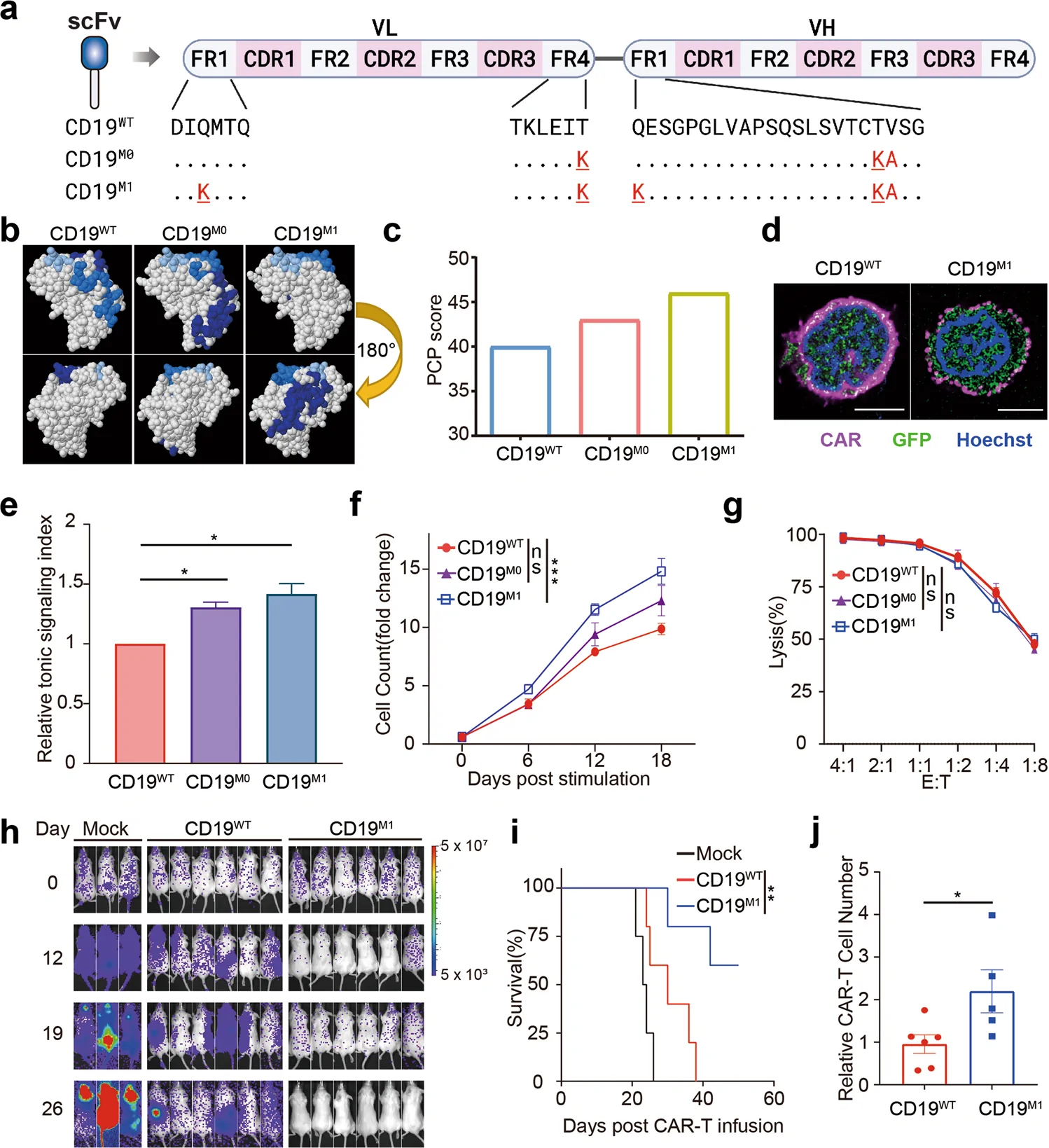
Incorporating PCPs into CARs containing 41BB costimulation domain with hypo tonic signaling promotes CAR-T persistence. **a** The sequences of optimized CD19.CAR variants. **b** The top three largest PCPs on modified CD19.CAR scFv surface displayed by the BindUP web server tool. Dark blue, the first-largest PCP; medium blue, the second-largest PCP; light blue, the third-largest PCP. **c** The calculation of PCP scores for the modified CD19.CAR scFvs. **d** Imaging analysis of the clustering of CD19^WT^ and CD19^M1^.CAR. Pink, CAR; green, CAR-IRES EGFP; blue, Hoechst. Scale bars, 5 μm. **e** The calculation of relative tonic signaling indexes for the modified CD19.CAR scFvs. **f** In vitro proliferation assay of WT and mutated CD19.CAR-T cells. **g** In vitro killing assay of WT and mutated CD19.CAR-T cells. **h** Representative bioluminescence images of tumor burden after CD19^WT^ and CD19^M1^.CAR-T infusion over time. **i** Survival curves for the tumor-bearing mice after CD19^WT^ and CD19^M1^.CAR-T infusion. **j** Number of CD19^WT^ and CD19^M1^.CAR-T cells in the spleen of tumor-bearing mice 1 month after CAR-T infusion. Data are presented as means ± SEM. Comparisons were determined using unpaired Student’s *t-*tests (**e**, **j)**, two-way analysis of variance (**f**, **g**), and survival analysis (**i**); *P < 0.05; **P < 0.01; ***P < 0.001; ns, not significant.

The tonic signaling of pre-BCR and pre-TCR is necessary for the survival and development of lymphocytes.^24^ Consistent with this, the inefficient antigen-independent tonic signaling was also reported to compromise the efficacy of CD22.41BBζ CAR-T cells against acute lymphoblastic leukemia.^6^ Considering the paradoxical effects of tonic signaling in effector functions of CAR-T cells against solid tumors and hematologic malignancies, we hypothesized that both too high and too low levels of tonic signaling would impair the CAR-T fitness. To exemplify the above, we utilized the CD19.CAR containing 41BB costimulation domain as a model with the lowest possible tonic signaling, as CD19.CAR exhibits the lowest tonic signaling index in our study and several studies have shown that the 41BB costimulation domain could further decrease the CAR tonic signaling strength compared to CD28 signaling domain.^4,20^ Interestingly, the distribution of positively charged residues on CD22.CAR surface is similarly uniform as that on CD19.CAR surface (Supplementary information, Fig. S5a–c), increasing the probability of inefficient tonic signaling intensity of CD19.CAR-T cells. To address this, we introduced PCPs into the CD19.CAR surface by mutating several uncharged amino acids in FRs into lysines (Fig. 5a). As expected, more positive residues and more expansive positive electric fields were observed on the surfaces of CD19^M0^ and CD19^M1^ than CD19^WT^ (Supplementary information, Fig. S5a, b, d). CD19^M1^ markedly enhanced the PCP scores and spontaneously formed CAR clustering, further entrenching the model that enhanced electrostatic interactions from PCPs are responsible for antigen-independent CAR clustering (Fig. 5b–d).

We then investigated whether the CAR-T antitumor function could be further improved in response to the raised PCPs of CD19.CAR. Unsurprisingly, CD19^M1^.CAR-T cells showed a higher tonic signaling index than CD19^WT^ (Fig. 5e). Since it is reported that a certain intensity of tonic signaling is required for cell proliferation and persistence of B lymphocytes and regulatory T cells expressing CAR,^25,26^ we compared T cell proliferation of CD19^WT^ and CD19^M1^.CAR-T cells. As shown in Fig. 5f, CD19^M1^.CAR-T cells demonstrated more robust proliferation than CD19^WT^.CAR-T cells in the proliferation assay in vitro.

The downstream signaling activities were also determined in CD19^WT^ and CD19^M1^.CAR-Ts. Similar to GD2.CAR, CD19.CAR expression could promote both MAPK and mTOR activities in T cells. Tuning up PCPs mainly enhanced the MAPK signaling level (Supplementary information, Fig. S5e–h). Together with the results shown in Supplementary information, Fig. S4k–n, our study revealed that the MAPK pathway might play a key role in mediating the effects of CAR tonic signaling. To investigate the downstream gene expression changes caused by CAR tonic signaling, we performed RNA-seq on different CAR-T cell samples. Along with the increase in PCP score, numerous genes associated with T cell stemness and quiescence, such as *TCF7* (encoding transcription factor TCF1), *IL7R*, *CCR7*, and *KLF2*, were downregulated, whereas the expressions of effector cell differentiation and exhaustion-related genes, including *PDCD1*, *TIGIT*, *GZMB*, and *FASL*, were upregulated (Supplementary information, Fig. S5i). These results suggest that CAR tonic signaling can regulate CAR-T cell stemness and exhaustion.

Finally, we sought to compare the antitumor function of CD19^WT^ and CD19^M1^.CAR-T cells in vitro and in vivo. CD19^M1^ and CD19^WT^.CAR-T cells showed comparable, if not identical, cytotoxicity ability and cytokine production level upon tumor cell coincubation, consistent with the notion that mutations within FRs have little effect on the CAR affinity against tumor antigen (Fig. 5g; Supplementary information, Fig. S5j–l).^27^ In contrast to similar antitumor function in vitro, CD19^M1^.CAR-T cells outperformed the CD19^WT^.CAR-T cells, achieving complete tumor eradication in vivo (Fig. 5h).^28^ Survival of CD19^M1^.CAR-T-treated mice was also significantly prolonged (Fig. 5i). Notably, CD19^M1^.CAR-T cells displayed a longer persistence than CD19^WT^.CAR-T cells in the periphery of the tumor-bearing mice post-infusion (Fig. 5j), likely contributed by the increased tonic signaling strength of PCP-introduced CAR. Our results demonstrated that introducing extra PCPs could restore inefficient CAR tonic signaling, leading to enhanced T cell persistence and antitumor activity of CAR-T therapy.

### PCP modulation within the FR of CAR scFv preserves its affinity and specificity

**Fig. 6:**
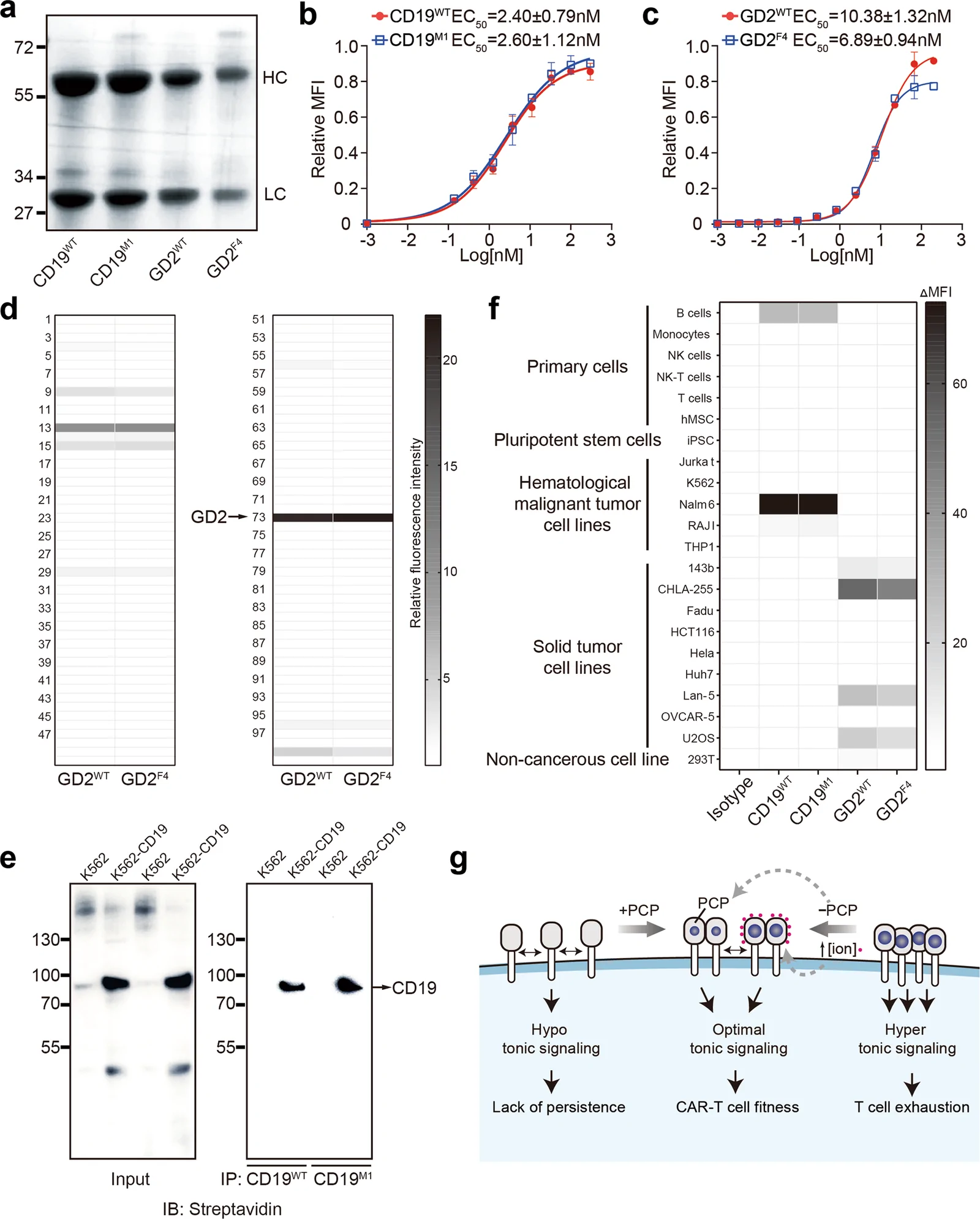
The antigen-binding affinities and specificities of WT and PCP-modified CARs. **a** Purified WT and PCP-modified antibodies stained with Coomassie Blue. HC, heavy chain; LC, light chain. **b**, **c** Antigen-binding activities of WT and PCP-modified antibodies measured by a cell-based FACS assay using antigen-positive cell lines. Nalm6 cells were used for CD19 antibodies (**b**), and GD2^+^Nalm6 cells were used for GD2 antibodies (**c**). EC_50_, half maximal effective concentration. Data are presented as means with 95% confidence intervals. **d** Binding activities of GD2^WT^ and GD2^F4^ antibodies against 100 synthetic glycans detected using the glycan array. **e** Biotin-labeled surface proteins on CD19^+^K562 cells enriched by CD19 antibodies. Cell surface proteins were biotinylated and immunoprecipitated using CD19^WT^ or CD19^M1^ antibodies, followed by detection via Streptavidin-HRP blot. WT K562 cells were included as a negative control. IP, immunoprecipitation. **f** Binding activities of WT and PCP-modified antibodies against a panel of human cells. MFI, Mean fluorescence intensity. **g** Schematic model illustrating rational improvement of tonic signaling strength and CAR-T cell fitness.

To elucidate whether the mutations we generated to modulate PCPs of CARs could affect their binding to the antigen, we purified GD2^WT^, GD2^F4^, CD19^WT^, and CD19^M1^ antibodies (Fig. 6a). We firstly compared the antigen-binding activity of WT antibodies with that of PCP-modified antibodies. Given that the antigen ganglioside GD2 is a type of glycolipid preferentially expressed on the surface of certain tumor cells and is, therefore, challenging for purification,^29^ we measured the binding activity of WT and mutated antibodies with their antigens by a cell-based FACS assay using antigen-positive cell lines. CD19^WT^ and CD19^M1^ antibodies were titrated for incubation with Nalm6 cells expressing CD19 on the cell surface. The EC_50_ of binding activity of CD19^WT^ and CD19^M1^ antibodies were 2.40 ± 0.79 nM and 2.60 ± 1.12 nM, respectively (Fig. 6b). Similarly, purified GD2^WT^ and GD2^F4^ antibodies were titrated to interact with GD2^+^NALM6 cells. These antibodies have a comparable EC_50_ (10.38 ± 1.32 nM vs 6.89 ± 0.94 nM) (Fig. 6c). These results suggest that CD19^M1^ and GD2^F4^ antibodies with mutations within the framework region largely preserve their antigen affinity.

Secondly, we examined the antigen specificity of WT and PCP-modified antibodies. The structural study has revealed that the GD2 antibody binds to the sugar moiety (not the lipid moiety) of GD2 ganglioside, which is exposed to the extracellular environment.^29^ Therefore, we tested the binding activities of GD2^WT^ and GD2^F4^ antibodies against 100 distinct synthetic glycans, including a panel of structurally related glycolipids. The glycan array analysis showed that the binding patterns of the GD2^WT^ and GD2^F4^ antibodies are almost identical (Fig. 6d). Two kinds of gangliosides are well recognized by GD2^WT^ and GD2^F4^ antibodies. The GD2 ganglioside (#73) has the strongest signal. The second strongest signal comes from #13, a kind of ganglioside containing *N*-Glycolylneuraminic acid (Neu5Gc) that is absent in humans.^30^ These results demonstrate that the GD2^WT^ and GD2^F4^ antibodies have the same antigen specificity.

It is reported that the CD19 antibody (clone#: FMC63) used in our study recognizes a conformational epitope of CD19 protein,^31^ as opposed to a linear epitope of CD19 protein that could be detected straightly by western blot. Therefore, we used the immunoprecipitation (IP) approach to test the antigen specificity. Firstly, we biotin-labeled the cell surface proteins on WT K562 cells, which do not express CD19, and those on CD19-transduced K562 cells. We then checked the specificity of CD19^WT^ and CD19^M1^ antibodies by immunoprecipitating biotin-labeled surface proteins, followed by blotting with streptavidin-HRP. Both the CD19^WT^ and CD19^M1^ antibodies could enrich one single primary band below the 100 kDa marker (Fig. 6e). It is known that CD19 has a theoretical molecular weight of 61 kDa, but an observed molecular weight is 95 kDa.^32^ Indeed, a single band with molecular weight of 95 kDa was only detectable in CD19-transduced K562 cell samples, not in WT K562 cell samples. Thus, these data indicate that this single band represents the CD19 protein. Our results suggest that both the CD19^WT^ and CD19^M1^ antibodies specifically recognize CD19 protein but not other surface proteins on K562 cells, indicating that they likely share the same antigen specificity.

Lastly, we further investigated the safety profile of WT- and PCP-modified CARs. We collected a panel of human cells consisting of six types of primary cells and fifteen cell lines derived from different tissues, as well as pluripotent stem cells. We then examined the reactivities of both WT and mutated antibodies against this human cell panel. We found that WT and PCP-modified antibodies shared similar, if not identical, recognition patterns against this human cell panel (Fig. 6f), indicating these PCP-modified antibodies do not cross-react with the tens of thousands of surface proteins expressed on 22 cell types in the tested human cell panel. Notably, no toxicity was observed in PCP-modified CAR-T cells (e.g., CD19^M1^ and GD2^F4^) in an animal model (Figs. 4j, 5h). Overall, our findings indicate that the safety profile of the original antibodies was unlikely to be changed by the mutations we created in the PCP areas.

Taken together, these findings imply that the point mutations we induced within the scFv framework region of CD19.CAR and GD2.CAR have minimal effects on CAR’s antigen affinity, antigen specificity, and safety profile.

## Discussion

Understanding why CAR-T cells generate signals autonomously and how signaling strength controls T cell antitumor activities will help us develop next-generation CAR-T cell therapy. Thus, we focus on the molecular mechanism underlying initiating and regulating CAR tonic signaling. Overall, our studies suggest a model for the association between CAR-T function fitness and CAR tonic signaling strength that is controlled by PCPs on the surface of the CAR antigen binding domain (Fig. 6g). In this model, inefficient tonic signaling leads to the lack of CAR-T persistence, whereas excessive tonic signaling results in CAR-T exhaustion. Consequently, optimal CAR-T cell fitness can be attained by fine-tuning tonic signaling through either modifying PCPs in CAR design or adjusting the ionic concentration during ex vivo culture.

The previous study implies that the instability of scFv determines CAR tonic signaling.^7^ However, a number of observations do not fit well with this scenario. First, in our study, a new CSPG4.CAR mutant with lower tonic signaling was generated by reducing the PCPs on the CAR surface. We calculated the change in free energy of this optimized CSPG4 scFv using Eris molecular suite, the same computing tool used in the previous study.^33^ Intriguingly, our optimized CSPG4.CAR had a dramatically elevated G value (ΔΔG > 10 kcal/mol), indicating a substantial loss in stability of scFv, yet exhibited a significantly lower tonic signal strength and greater efficacy (Supplementary information, Fig. S4s–u). Second, among nine mutations generated to eliminate the CSPG4.CAR tonic signaling in the previous study, three uncharged amino acids were mutated into positively charged lysines and a negatively charged glutamic acid was substituted with an uncharged amino acid, indicating the importance of the electronic property of CARs surface and favoring our PCP model.^7^ Third, misfolded or unstable membrane proteins are often targeted for degradation.^34^ However, the modified CAR expression was largely unaffected when the tonic signaling strength was altered by introducing or removing PCPs on the CAR surface. Lastly, when ten commonly used CARs were examined, no significant correlation was observed between the instability of scFv and CAR tonic signaling index (Supplementary information, Fig. S2e), demonstrating that scFv instability is not the primary determinant for CAR tonic signaling.

In contrast, several lines of evidence support our theory that PCPs on scFv determine CAR tonic signaling strengths. First, previous research and our present study suggest that the CAR signaling strength might depend on the scFv region of CARs. Interestingly, PCPs on antibody surfaces are demonstrated to be correlated with the aggregation and nonspecific binding of antibodies,^35–37^ and charged residue substitutions in FRs could reduce aggregation potential and increase the developability of these antibodies.^38^ Second, to improve the affinity of GD2-binding, one single mutation (E101K) was introduced in the CDR3 of the original scFv (clone#14g2a), which directly contacts the antigen.^39^ The resulted GD2 CAR variant displayed higher antigen-independent tonic signaling strengths and more severe exhaustion phenotypes,^5,40^ likely due to enhanced PCP score resulting from mutating one negatively glutamic acid to positively charged lysine. Third, it is reported that the high tonic signaling GD2.CAR-T cells expanded ex vivo in a medium conditioned with the addition of carnosine, a biogenic amine modestly increasing the incubation pH, had superior potency in clearing tumors,^22^ which could be well explained by our model, since increasing the pH value in the medium can reverse the surface charge of proteins substantially from positive to negative. Lastly, inspired by these findings, we sought to reduce the PCPs on high tonic signaling CARs, such as GD2 and CSPG4.CARs, and we discovered that these optimized CARs with lower PCP scores minimized tonic signaling, ameliorated exhaustions, and ultimately improved efficacies (Fig. 4; Supplementary information, Fig. S4).

Electrostatically driven interactions between proteins contribute to the aggregation and solubility of proteins.^41^ Proteins with positively charged surface patches are prone to aggregate, whereas proteins carrying negatively charged surfaces are more soluble.^42^ This phenomenon may be correlated with the interactions between PCPs and anions,^37^ as the addition of polyanions could promote the stability of proteins with PCPs.^43^ These observations may also suggest a potential mechanism for spontaneous CAR clustering. Unlike antibodies in the solution, CARs with PCPs expressed on the T cell surface have possible electrostatic interactions with negatively charged phospholipids in the cell membrane. A potential candidate is the negatively charged phosphatidylserine (PS). PS is normally restricted to the inner plasma-membrane leaflet, and this lipid asymmetry is lost during apoptosis. However, PS exposure on the cell membrane is also observed in either antigen-activated T lymphocytes^44^ or a subpopulation of T cells.^45^ Exposed PS on T cells colocalizes with lipid rafts at the immunologic synapse. Blocking exposed PS could significantly inhibit T cell cytokine production and T cell migration.^44,45^ Furthermore, negatively charged PS on cell membrane could capture and slowly release cytokines (e.g., IFN-γ) by binding their positively charged regions.^46^ Therefore, a potential electrostatic attraction may exist between CAR PCPs and exposed PS on the T cell membrane, which would facilitate antigen-independent CAR clustering.

A reproducible ex vivo expansion of engineered T cells with consistently high antitumor function is vital for clinical cell therapy. High tonic signaling CARs, e.g., GD2, drives T cells to exhaustion during the ex vivo expansion. An optimal setting for expanding these exhaustion-prone CAR-T cells is urgently needed. Here, we found that increasing the ionic strength in the culture medium for ex vivo expansion remarkedly reduces the CAR tonic signaling, but not antigen-induced signaling, which is a convenient approach to improve CAR-T efficacy against solid tumors.

CAR-T in vivo persistence is a key factor in determining CAR-T function,^47^ which relies on an appropriate strength of tonic signaling.^14^ Two types of CARs, CD19 and CD22, currently being used to target hematologic malignancies, exhibit weak tonic signals. It was reported that knocking out TCR, a typical strategy for generating universal CAR-T, could abate CD19.CAR-T tonic signaling and CAR-T persistence,^48^ whereas improving tonic signaling could benefit CD22.CAR-T function and clinical efficacy.^6^ Given the lack of a convenient system for modulating CAR-T tonic signaling strength, our novel approach of introducing PCPs on the CAR surface has critical implications for the clinical translation of CAR-T therapy against hematologic malignancies.

Overall, our study demonstrated that autonomous CAR oligomerization mediated by the uneven distribution of charge density on the surface of the CAR antigen binding domain induces CAR tonic signaling. We also developed a bioinformatic tool (PCP score) to quantify the positively charged patches on the CAR surface, as a predictor of the CAR tonic signaling. To improve CAR-T fitness, the strength of CAR tonic signaling could be rationally fine-tuned via altering the effect of PCP by genetic mutation or by adjusting the ionic strength during ex vivo expansion. According to our findings, the PCP score that generates optimal CAR signaling might be around 46–56 (the PCP score of CD19^M1^ and GD2^F4^, respectively). Notably, the optimal signaling for various CARs may be context-dependent, and the precise definition of optimal CAR signaling needs to be determined in future studies.

## Materials and methods

### Reagents and antibodies

Cell culture medium and the supplements, including FBS, were purchased from Thermo Scientific. NaCl (S5886) was from Sigma. Hoechst 33342 (H1399) was from Invitrogen. D-luciferin (122799) was from PerkinElmer. Human T-Activator CD3/CD28 Dynabeads (11132D) were from Life Technologies. Recombinant human IL-2 (GMP-CD66) was from Novoprotein. The following antibodies were used for FACS: anti-CD69-PE (1:800, Biolegend, 310906), anti-CD25-PerCP-Cy5.5 (1:800, Biolegend, 302625), anti-ICOS-PE-CY7 (1:800, Biolegend, 313520), anti-Myc-Alexa Fluor 647 (1:1000, Cell Signaling Technology, 2233 S), anti-PD-1-PE (1:800, Biolegend, 367404), anti-LAG-3-PE-Cy7 (1:800, eBioscience, 25-2239-42), anti-TIM-3-PerCP-Cy5.5 (1:800, Biolegend, 345016), anti-CD62L-PE (1:1000, Biolegend, 304806), anti-CD45RA-PE-Cy7 (1:1000, Biolegend, 304125), anti-Tox/Tox2-Alexa Fluor 647 (1:50, Cell Signaling Technology, 82473 S), anti-TCF1/TCF7-Pacific Blue (1:50, Cell Signaling Technology, 9066 S), anti-CD8a-Brilliant Violet 605 (1:800, Biolegend, 300936). The following antibodies were used for Confocal microscopy: anti-Myc (1:800, Cell Signaling Technology, 2276), anti-mIgG-Alexa Fluor 647 (1μg/mL, Jackson, 115-606-072). The anti-Streptavidin-HRP antibody (1:2000, Cell Signaling Technology, 3999 S) was used for western blot. IL-2 ELISA kit (550611), IFN-γ ELISA kit (550612), and TNF-α ELISA kit (550610) were from BD biosciences. The Glycan Array kit was from Raybiotech (GA-Glycan-100-1).

### Cell lines

Lenti-X 293 T, Huh-7, 143B, FaDu, HeLa and K562 cell lines were purchased from TaKaRa and ATCC. Jurkat and Raji B cell lines were provided by Author Weiss at University of California, San Francisco. NALM-6, GD2^+^ CHLA-255 and LAN-1 cell lines, CSPG4^+^ CNE-2, and THP-1 cell lines were kindly gifted from Jie Sun at Zhejiang University, Yang Xu at Southern University of Science and Technology, Xiaoshen Wang at Fudan University, Xianmin Song at Shanghai Jiao Tong University, respectively. U2OS, HCT116, iPSC, and hMSC were kindly provided by Zhaobo Lin at ShanghaiTech University. As previously described, tumor cell lines were engineered to express human CD19, GD2, or/and firefly luciferase for killing and mouse experiments, respectively. The adherent cells were cultured in DMEM medium while suspension cells were maintained in RPMI-1640 medium, both of which were supplemented with 10% FBS, 100 U/mL penicillin and 100 mg/mL streptomycin.

TCR-knockout Jurkat cell line was created in our lab using the CRISPR/Cas9 system. Guide RNAs targeting TRAC and TRBC1 were designed using Benchling (http://Benchling.com) and cloned into pX330 vector (targeting TRAC, gagaatcaaaatcggtgaat; targeting TRBC1, ggctctcggagaatgacgag). Jurkat cells were electroporated using the Gene Pulser Xcell Electroporation system from Bio-Rad, followed by the manufacturer’s specifications. TCR-knockout Jurkat cells were sorted using FACSAria III cell sorter (BD).

### Computational analysis

We used the protein structure homology-modeling server SWISS-MODEL (https://swissmodel.expasy.org) to generate the 3D conformation of CAR scFvs,^49^ and analyzed their characteristics of electrostatic potential using the Swiss-PdbViewer software.^50^ Electrostatic surfaces of CAR scFvs were calculated using APBS^51^ and displayed in UCSF Chimera v1.14.^52^ For stability quantifications of CAR scFvs, a protein-sol heatmap software was used.^53^ The net charge (taking into account both positive and negative charges) of CAR scFvs was also calculated using the protein-sol heatmap software, and the net charge is divided by total number of surface amino acids. The results were shown as positive/negative charge per amino acid. To display and calculate the three largest positive electrostatic patches of CAR scFvs, the BindUp web server was used.^54^ Framework regions of CAR scFvs were determined using the IMGT/V-QUEST tool, and the alternative framework sequences were obtained using the IMGT databases.^55^

### CAR construction and lentivirus production

The generations of CAR plasmids and lentivirus were conducted as reported in our previous work.^1^ In short, each CAR comprised of a specific scFv, a CD28/41BB costimulatory domain, a CD3ζ signaling domain, and an IRES-EGFP cassette was cloned into a modified PHR vector with the hEF1α promoter. Point mutations and replacements of framework regions for CARs were performed via PCR and Gibson assembly cloning. For image analysis, a myc-tag was fused into the CAR construct. Lentivirus supernatants were generated in Lenti-X 293 T cells with the CAR plasmid and viral packaging plasmids.

### Transduction and expansion of human T cells

Human peripheral blood mononuclear cells from healthy donors were cultured in X-VIVO medium (Lonza, 04-418Q) supplemented with 2% FBS, 100 U/mL penicillin, 100 mg/mL streptomycin, 0.292 mg/mL glutamine, and 200 U/mL recombinant hIL-2. Before transduction, T cells were stimulated with Human T-Activator CD3/CD28 Dynabeads for 24 h firstly. On the next day, T cells were incubated with the virus for 18 h before medium replacement. Medium containing hIL-2 was refreshed every 2–3 days. Dynabeads were removed on day 4. In certain cases, sodium chloride was added into the medium after transduction. Cells used for experiments were harvested about 2 weeks after transduction.

### Flow cytometry analysis

For cell-surface staining, cells were incubated with antibodies at 4 °C for 30 min in the dark. For intracellular staining, cells were fixed and permeabilized by the FoxP3 Transcription Factor buffer set (ThermoFisher Scientific, 00-5523-00). Antibodies were incubated for 1 h at room temperature. In most cases, Zombie Violet Fixable viability Kit was used to exclude dead cells. Samples were acquired on LSRFortessa and analyzed with FlowJo v.10 software. For cell sorting, cells were stained as described above and sorted using the flow cytometry FACSAria III system. Post-sort purity was over 95%.

### Calculation of tonic signaling index and exhaustion score

To measure the tonic signaling index, Jurkat cells were transduced by CAR-expressing virus for 3 days, and these cells were with CD69. After GFP-positive cells were gated, the mean fluorescence intensity (MFI) of CD69-PE and MFI of GFP in these cells were measured. CAR tonic signaling index was calculated using the following formula:

$${{{{{{{\mathrm{CAR}}}}}}}}\;{{{{{{{\mathrm{tonic}}}}}}}}\;{{{{{{{\mathrm{signaling}}}}}}}}\;{{{{{{{\mathrm{index}}}}}}}} = {{{{{{{\mathrm{MFI}}}}}}}}^{{{{{{{{\mathrm{CD}}}}}}}}69}/{{{{{{{\mathrm{MFI}}}}}}}}^{{{{{{{{\mathrm{GFP}}}}}}}}}$$

To calculate the exhaustion score, primary T cells were transduced by CAR-expressing virus for 10 days, and GFP-positive cells were gated. The surface expression of PD-1, Lag-3 and Tim-3 in these GFP-positive cells was determined by FACS. CAR exhaustion score was assessed using the following formula:

$${{{{{{{\mathrm{CAR}}}}}}}}\;{{{{{{{\mathrm{exhaustion}}}}}}}}\;{{{{{{{\mathrm{score}}}}}}}} = \,	({{{{{{{\mathrm{MFI}}}}}}}}^{{{{{{{{\mathrm{PD}}}}}}}} {\mbox{-}} 1}/{{{{{{{\mathrm{MFI}}}}}}}}^{{{{{{{{\mathrm{GFP}}}}}}}}} + {{{{{{{\mathrm{MFI}}}}}}}}^{{{{{{{{\mathrm{Lag}}}}}}}} {\mbox{-}} 3}/{{{{{{{\mathrm{MFI}}}}}}}}^{{{{{{{{\mathrm{GFP}}}}}}}}} \\ 	 + {{{{{{{\mathrm{MFI}}}}}}}}^{{{{{{{{\mathrm{Tim}}}}}}}} {\mbox{-}} 3}/{{{{{{{\mathrm{MFI}}}}}}}}^{{{{{{{{\mathrm{GFP}}}}}}}}})/3$$

### RNA-seq

RNA-seq was performed based on the SMART-Seq2 protocol with modifications.^56^ Briefly, 2 × 10^6^ CD8^+^ CAR-T cells were sorted and the total mRNA was extracted. 100 ng total RNA was mixed with TSO oligo-dT primer (5′-AAGCAGTGGTATCAACGCAGAGTACT_30_VN-3′). Hybridized RNA was reverse transcribed using Template Switching RT Enzyme Mix (M0466S, NEB), and cDNA was amplified with 8 cycles by Q5 High-Fidelity DNA Polymerase (M0491S, NEB). After PCR pre-amplification, cDNA was purified by AMPure XP beads (A63880, Beckman Coulter) and subjected to Tn5 Tagmentation. Final RNA-seq libraries were generated by PCR amplification and sequenced on an Illumina Novaseq system. Pair-end data were aligned to human genome assembly GRCh37/hg19 using STAR v2.7.5a.^57^ Mapped reads were counted for each gene using the HTSeq v0.12.4.^58^ Individual count files were merged by an in-house R script and normalized using the DESeq2 R package. Advanced Heatmap Plots to visualize specific gene expressions were performed using the OmicStudio tools at https://www.omicstudio.cn.

### Confocal microscopy

For imaging analysis of CAR clustering, Jurkat cells bearing indicated CARs were fixed with 4% paraformaldehyde and permeabilized using 0.1% Triton X-100 in PBS. Cells were blocked using 2% BSA in PBS before staining. CAR clusters were labeled using anti-myc antibody and followed by anti-mAb- Alexa Fluor 647, while nuclear was stained with Hoechst 33342. Images were observed and captured using Nikon TI2-E CSU W1 microscope and analyzed with the ImageJ software.

### In vitro cytotoxicity assay

Targeted tumor cells were engineered to stably express the firefly luciferase firstly. The luciferase-expressing tumor cells were seeded at 5 × 10^4^ cells/well in 96-well plates and incubated with various numbers of CAR-T cells at multiple E:T ratios for 24 h at 37 °C. Subsequently, the co-culture system of each well was transferred to a specific luminometer plate, and 0.15 mg D-luciferin was added afterwards. PerkinElmer Enspire was used to observe the luminescence intensity of each well immediately. The killing efficacy was calculated by normalizing luminescence intensity of coculture wells to that of tumor cell monoculture.

### Cytokine production assay

CAR-T cells for experiments were seeded into 96-well plates (10^5^ cells/well). Various amounts of targeting tumor cells were added into the wells at indicated E/T ratios. After a 24 h incubation, supernatants were collected and cytokines were measured using the ELISA kits following standardized protocols provided by the kit manual.

### In vitro proliferation assay

The proliferative ability of CAR-T cells was assessed as in our previous work.^47^ Briefly, 1 × 10^6^ CAR-T cells were stimulated by irradiated tumor cells (1:1 E:T) for 3 days after a full rest without hIL-2. Cell number was counted every other day by Trypan Blue dye exclusion, and the cell culture density was kept at 1 × 10^6^ T cells per mL with fresh medium meanwhile.

### Mouse experiments

As described in our previous study,^47^ 6- to 8-week-old NOD/SCID/Il2rg-null (B-NSG) mice from Biocytogen were used in the mouse experiments. For anti-GD2 xenograft experiments using Nalm6-GD2, mice were inoculated with 5 × 10^5^ luciferase-expressing tumor cells 4 days before adoptive cell transfer of 2 × 10^6^ CAR-T or non-transduced T cells intravenously. For anti-CD19 xenograft experiments using Nalm6, mice were inoculated with 5 × 10^5^ luciferase-expressing tumor cells 4 days before adoptive cell transfer of 1 × 10^6^ CAR-T or non-transduced T cells intravenously. Tumor progression was monitored by bioluminescence imaging using the IVIS spectrum imaging system (PerkinElmer). Quantitative values were acquired using LivingImage Software (PerkinElmer). Mice were euthanized when signs of excessive tumor burden were observed, and survival curve was made accordingly. Spleen was collected to evaluate the persistence of CAR-T cells using FACS at the indicated time point. All animal experiments were approved by our animal ethical committee.

### Antibody expression and purification

WT and PCP-modified antibodies were cloned into pCMV plasmid fused with human IgG1 constant region and transfected into 293 T cells with polyethyleneimine. Antibody was purified by protein A beads from the cell culture supernatant and eluted by 50 mM Gly-HCl (pH 3.0), neutralized with 1 M Tris (pH 8.0). Antibodies were then changed into PBS buffer concentrated with 50 kDa Amicon Ultra-4 tube (Millipore, UFC805024) and protein concentration was quantified using nanodrop. About 4 μg antibody was used for protein purity test by SDS-PAGE gel electrophoresis using Coomassie Blue.

### Glycan array

Glycan array analysis was performed according to the manufacturer’s instruction manual. Briefly, GD2^WT^ and GD2^F4^ antibodies were labeled with biotin after dialysis. Glycan microarray slides were dried and blocked before incubating with biotin-labeled antibodies overnight at 4 °C. After washing with the buffer supplied within the kit, the Cy3 equivalent dye-conjugated streptavidin was added into each well and incubated at room temperature for 1 h in the dark. Finally, microarray slides were washed, dried, and detected using an InnoScan 300 Microarray Scanner (Innopsys) at OD 532 nm and analyzed with the software provided by the manufacturer.

### Biotin labeling and Immunoprecipitation

WT and CD19-transduced K562 cells were harvested and biotinylated using Sulfo-NHS-SS-biotin (APExBIO, #A8005) at 1 mg/mL for 15 min, followed by lysing in RIPA lysis buffer supplemented with protease inhibitors and incubated on ice for 30 min. Then centrifugation was performed to get clear debris at 14000× *g*, 10 min, 4 °C. 1/20 of lysate was used as input and the rest of the lysate was incubated with 3 μg purified CD19^WT^ or CD19^M1^ antibody on a rotator at 4 °C overnight. Antibody-conjugated lysates were incubated with protein A beads on a rotator at 4 °C for 2 h. Beads were then washed with RIPA buffer three times and reserved for streptavidin blot analysis.

### Statistical analysis

All data were presented as means ± SEM if not otherwise indicated. Analyses of differences were determined using unpaired Student’s *t*-test, two-way analysis of variance, or survival analysis. Statistical analyses were performed using GraphPad Prism (GraphPad Software, Inc.). All *P* values presented were two-tailed, and *P* < 0.05 was considered statistically significant.

## Supplementary information

Fig. S1

Fig. S2

Fig. S3

Fig. S4

Fig. S5
