# Supplementary material for: Tuning charge density of chimeric antigen receptor optimizes tonic signaling and CAR-T cell fitness
Source: Cell Res. 2023 Mar 8;33(5):341–54. doi: 10.1038/s41422-023-00789-0 (PMC10156745; doi:10.1038/s41422-023-00789-0)
Supplement: Supplementary file 1 — Fig. S1 [file 41422_2023_789_MOESM1_ESM.pdf]

Figure S1

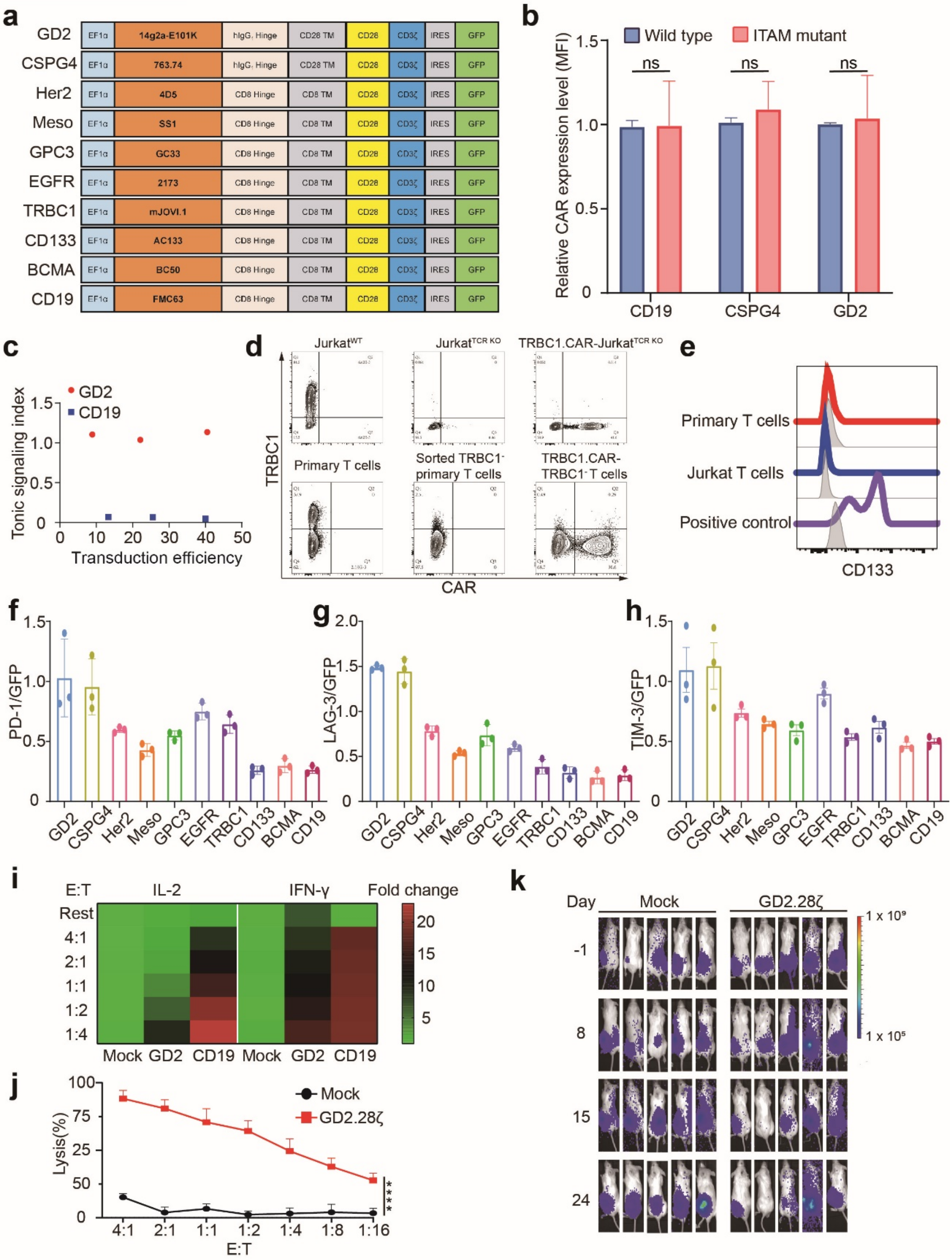

a
GD2
CSPG4
Her2
Meso
GPC3
EGFR
TRBC1
CD133
BCMA
CD19
EF1α
14g2a-E101K
763.74
4D5
SS1
GC33
2173
mJOVI.1
AC133
BC50
FMC63
CD8 Hinge
CD28 TM
CD8 TM
CD28
CD3ζ
IRES
GFP
b
Wild type
ITAM mutant
ns
Relative CAR expression level (MFI)
CD19
CSPG4
GD2
c
GD2
CD19
Tonic signaling index
Transduction efficiency
d
Jurkat^WT
Jurkat^TCR KO
TRBC1.CAR-Jurkat^TCR KO
Primary T cells
Sorted TRBC1- primary T cells
TRBC1.CAR-TRBC1- T cells
TRBC1
CAR
e
Primary T cells
Jurkat T cells
Positive control
CD133
f
PD-1/GFP
g
LAG-3/GFP
h
TIM-3/GFP
i
E:T
IL-2
IFN-γ
Fold change
Rest
4:1
2:1
1:1
1:2
1:4
Mock
GD2
CD19
j
Mock
GD2.28ζ
Lysis(%)
E:T
****
k
Day
Mock
GD2.28ζ
1 x 10⁹
1 x 10⁵

Figure S1. T cells incorporating many commonly used CARs express exhaustion markers and exhibit dysfunctional phenotypes.

(**a**) The structures of the CARs used in this study.

(**b**) Relative CAR expression levels after ITAM mutation in CD19, CSPG4, and GD2 CAR-T cells.

(**c**) Correlation between the transduction efficiency and tonic signaling index.

(**d**) Expressing TRBC1.CAR on TCR knockout Jurkat cells and sorted $TRBC1^-$ primary T cells.

(**e**) Surface expression of CD133 on Jurkat T cells and primary T cells. A CD133 transfected 293T cell sample was included as the positive control.

(**f-h**) The GFP-normalized PD-1, LAG-3, and TIM-3 expression levels of ten commonly used CARs.

(**i**) Cytokine secretion assay of GD2 and CD19 CAR-T cells after activation. Indicated CAR-T cells were harvested and co-incubated with their target tumor cells at the indicated E:T ratio. The levels of IL-2 and IFN-γ were detected by ELISA.

(**j**) In vitro killing assay against $GD2^+$ neuroblastoma cell line CHLA-255 of GD2 CAR-T cells.

(**k**) In vivo killing assay against $GD2^+$ neuroblastoma cell line CHLA-255 of GD2 CAR-T cells.

Data are presented as means ± SEM; Comparisons were determined using unpaired student's t-tests (b) and two-way analysis of variance (j); **** $P < 0.0001$; ns not significant.
