# Supplementary material for: Tuning charge density of chimeric antigen receptor optimizes tonic signaling and CAR-T cell fitness
Source: Cell Res. 2023 Mar 8;33(5):341–54. doi: 10.1038/s41422-023-00789-0 (PMC10156745; doi:10.1038/s41422-023-00789-0)
Supplement: Supplementary file 2 — Fig. S2 [file 41422_2023_789_MOESM2_ESM.pdf]

Figure S2

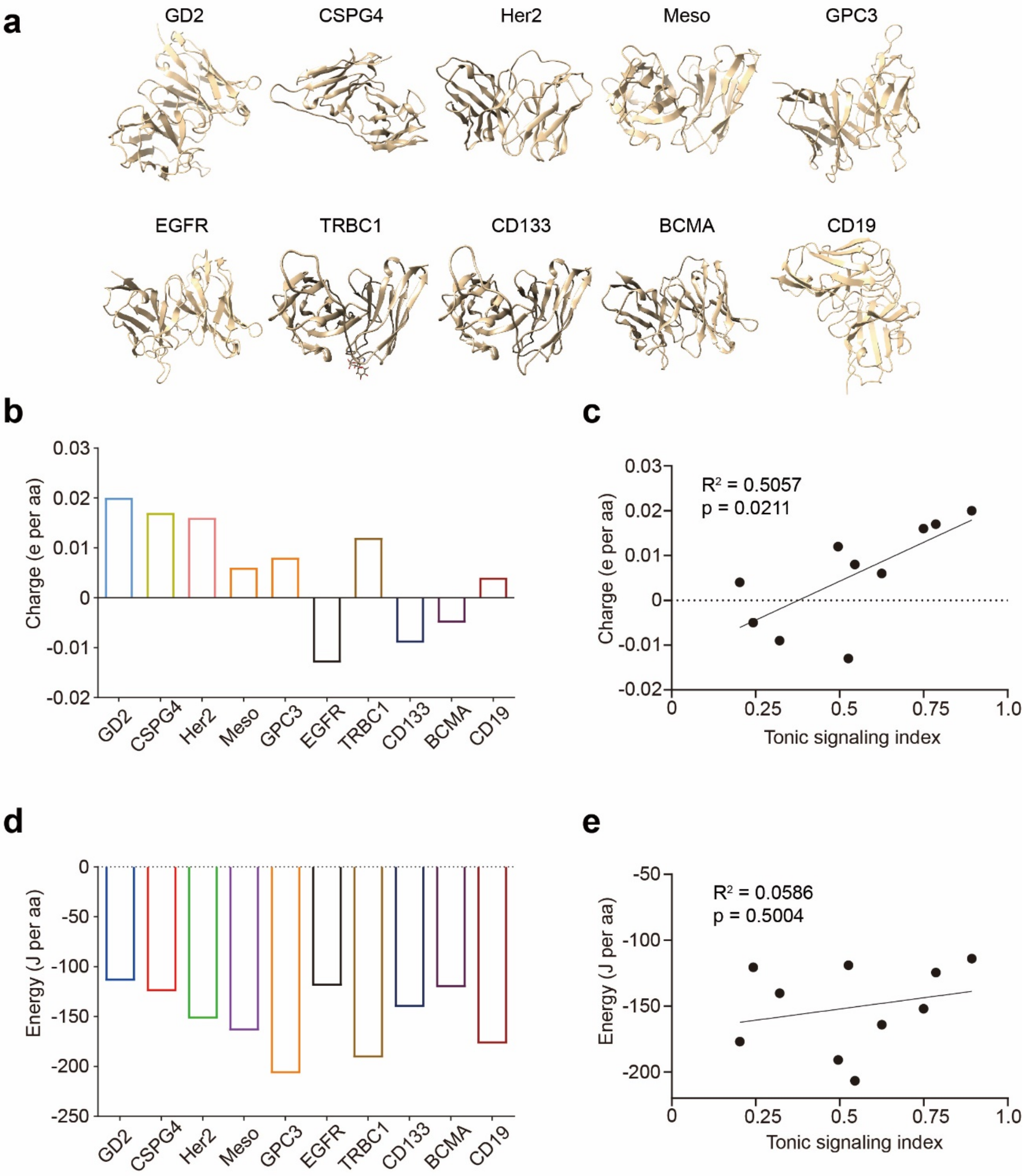

Figure S2. Biophysical properties of the ten CAR scFvs and their potential correlation with CAR-T tonic signaling indexes.

(**a**) Model building for the ten CAR scFvs using SWISS-MODEL.

(**b**) Net amino acid charges of the ten CAR scFvs at 0.15M Ionic strength and PH 7.5 calculated using the Protein–Sol webserver.

(**c**) Correlation between the net amino acid charge and the tonic signaling index in these CAR-T cells, assessed by the Pearson method.

(**d**) Energies of the ten CAR scFvs at 0.15M Ionic strength and PH 7.5 calculated using the Protein–Sol webserver. The energy is given in Joules per amino acid.

(**e**) Correlation between the energy and the tonic signaling index in these CAR-T cells, assessed by the Pearson method.
