# Supplementary material for: Tuning charge density of chimeric antigen receptor optimizes tonic signaling and CAR-T cell fitness
Source: Cell Res. 2023 Mar 8;33(5):341–54. doi: 10.1038/s41422-023-00789-0 (PMC10156745; doi:10.1038/s41422-023-00789-0)
Supplement: Supplementary file 3 — Fig. S3 [file 41422_2023_789_MOESM3_ESM.pdf]

Figure S3

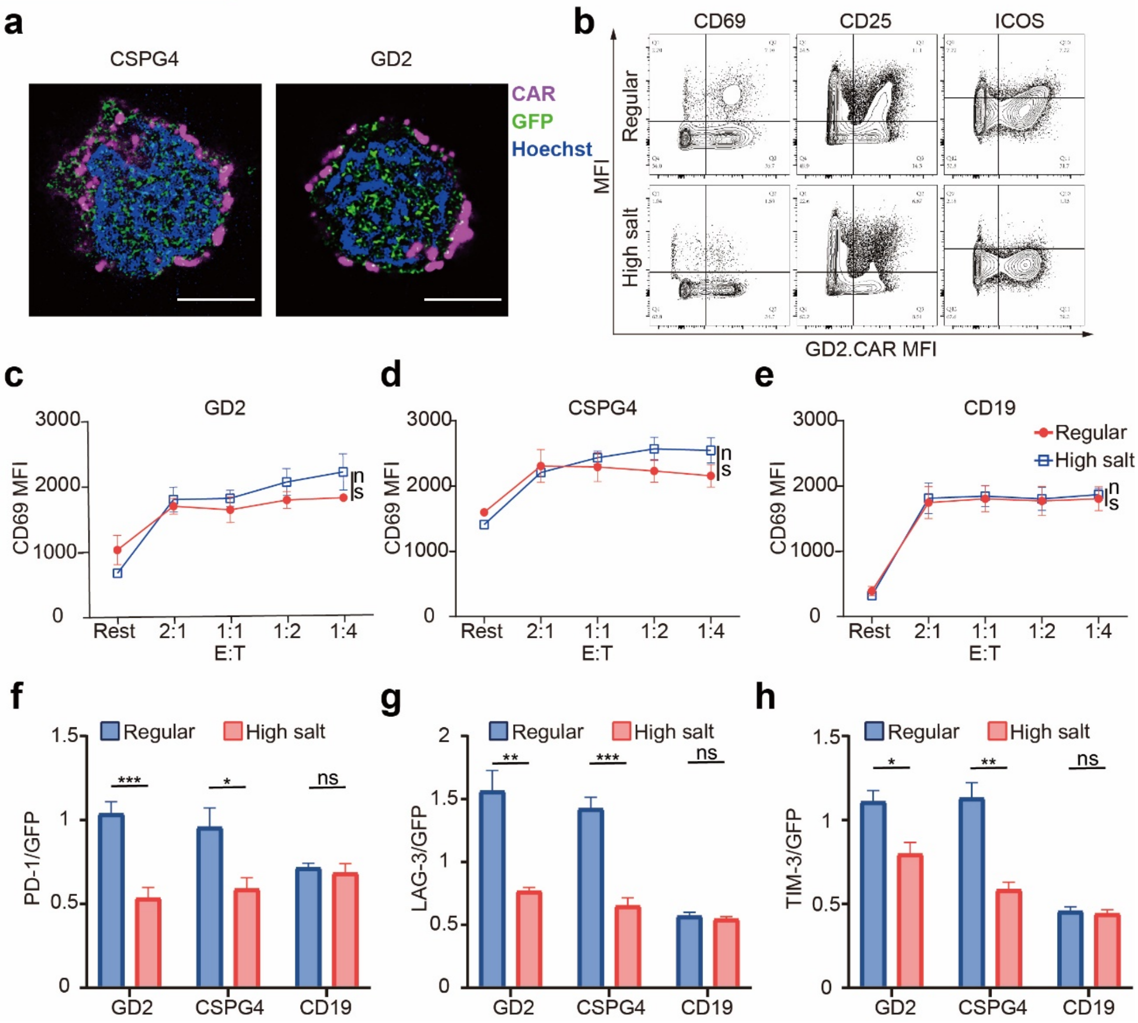

Figure S3. High tonic CAR-T cells cultured from high-salt medium exhibit reduced tonic signaling and less exhausted phenotype.

(**a**) Imaging analysis of CAR clustering of Jurkat T cells expressing ITAM mutant CSPG4 or GD2 CARs. Pink: CAR; green: CAR-IRES EGFP; blue: Hoechst. Scale bars, 5μm.

(**b**) Representative FACS plot of CD69, CD25, and ICOS expression of GD2 CAR-T cells cultured in either regular or high-salt medium.

(**c-e**) Jurkat T cells expressing indicated CAR were cultured in either regular or high-salt medium and CD69 expressions upon tumor antigen engagement were shown.

(**f-h**) Primary T cells expressing indicated CAR were cultured in either regular or high-salt medium and exhaustion markers, including PD-1, LAG-3, and TIM-3, normalized by GFP expression were shown.

Data are presented as means ± SEM; Comparisons were determined using two-way analysis of variance (c-e) and unpaired student's t-tests (f-h); * *P* < 0.05; ***P* < 0.01; ****P* < 0.001; ns not significant.
