# Supplementary material for: Tuning charge density of chimeric antigen receptor optimizes tonic signaling and CAR-T cell fitness
Source: Cell Res. 2023 Mar 8;33(5):341–54. doi: 10.1038/s41422-023-00789-0 (PMC10156745; doi:10.1038/s41422-023-00789-0)
Supplement: Supplementary file 4 — Fig. S4 [file 41422_2023_789_MOESM4_ESM.pdf]

Figure S4

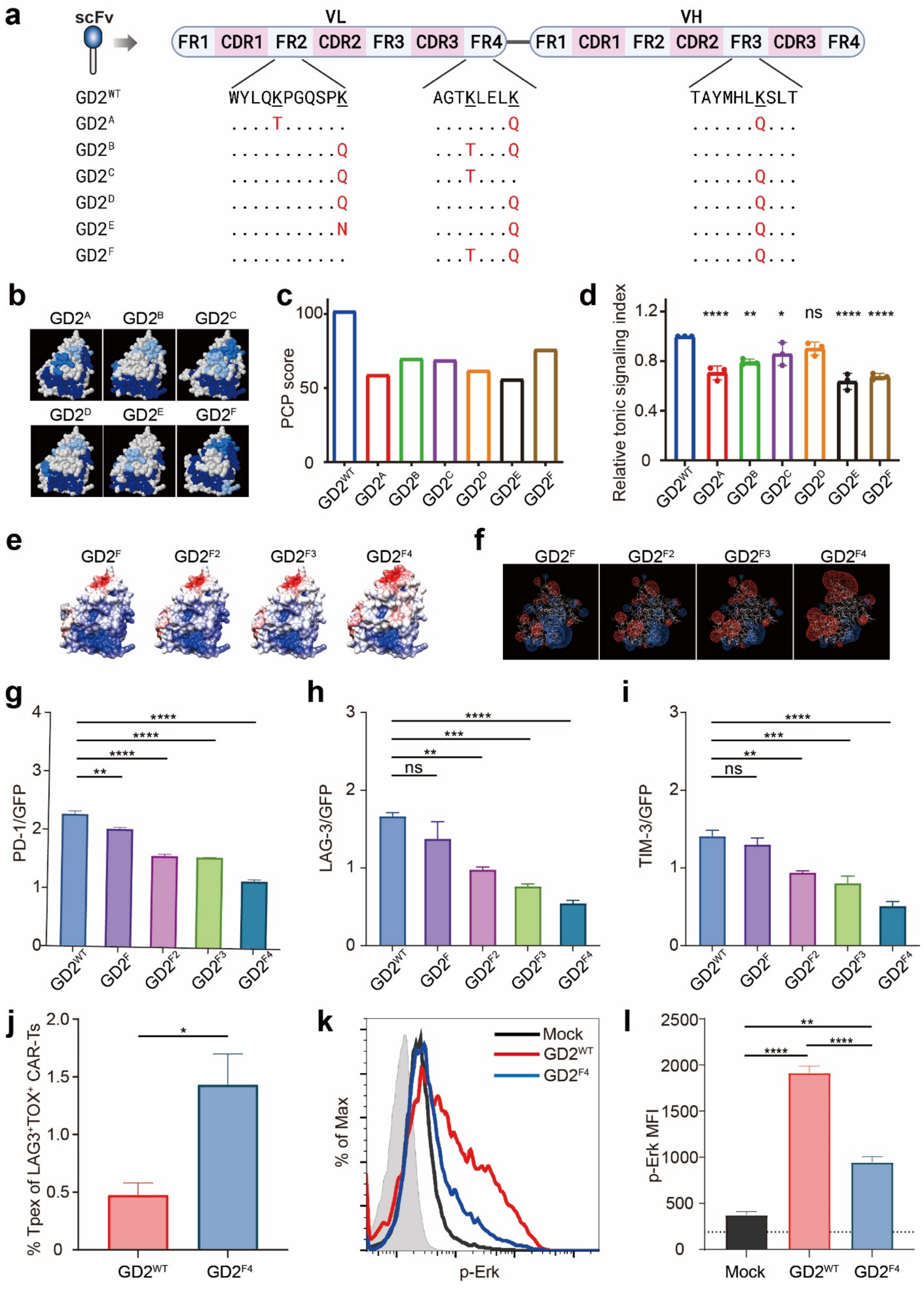

a
scFv
VL
VH
FR1 CDR1 FR2 CDR2 FR3 CDR3 FR4
FR1 CDR1 FR2 CDR2 FR3 CDR3 FR4
GD2WT WYLQKPGQSPK AGTKLELK TAYMHLKSLT
GD2A ....T...... ....Q ......Q...
GD2B ....Q ...T...Q ....
GD2C ....Q ...T.... ......Q...
GD2D ....Q ....Q ......Q...
GD2E ....N ....Q ......Q...
GD2F ....T...Q ......Q...
b
GD2A GD2B GD2C
GD2D GD2E GD2F
c
PCP score
100
50
0
GD2WT GD2A GD2B GD2C GD2D GD2E GD2F
d
Relative tonic signaling index
1.2
0.8
0.4
0
**** ** * ns **** ****
GD2WT GD2A GD2B GD2C GD2D GD2E GD2F
e
GD2F GD2F2 GD2F3 GD2F4
f
GD2F GD2F2 GD2F3 GD2F4
g
PD-1/GFP
4
3
2
1
0
**
****
****
****
GD2WT GD2F GD2F2 GD2F3 GD2F4
h
LAG-3/GFP
3
2
1
0
ns
**
***
****
GD2WT GD2F GD2F2 GD2F3 GD2F4
i
TIM-3/GFP
3
2
1
0
ns
**
***
****
GD2WT GD2F GD2F2 GD2F3 GD2F4
j
% Tpex of LAG3+TOX+ CAR-Ts
2.0
1.5
1.0
0.5
0
*
GD2WT GD2F4
k
Mock
GD2WT
GD2F4
% of Max
p-Erk
l
p-Erk MFI
2500
2000
1500
1000
500
0
**
****
****
Mock GD2WT GD2F4

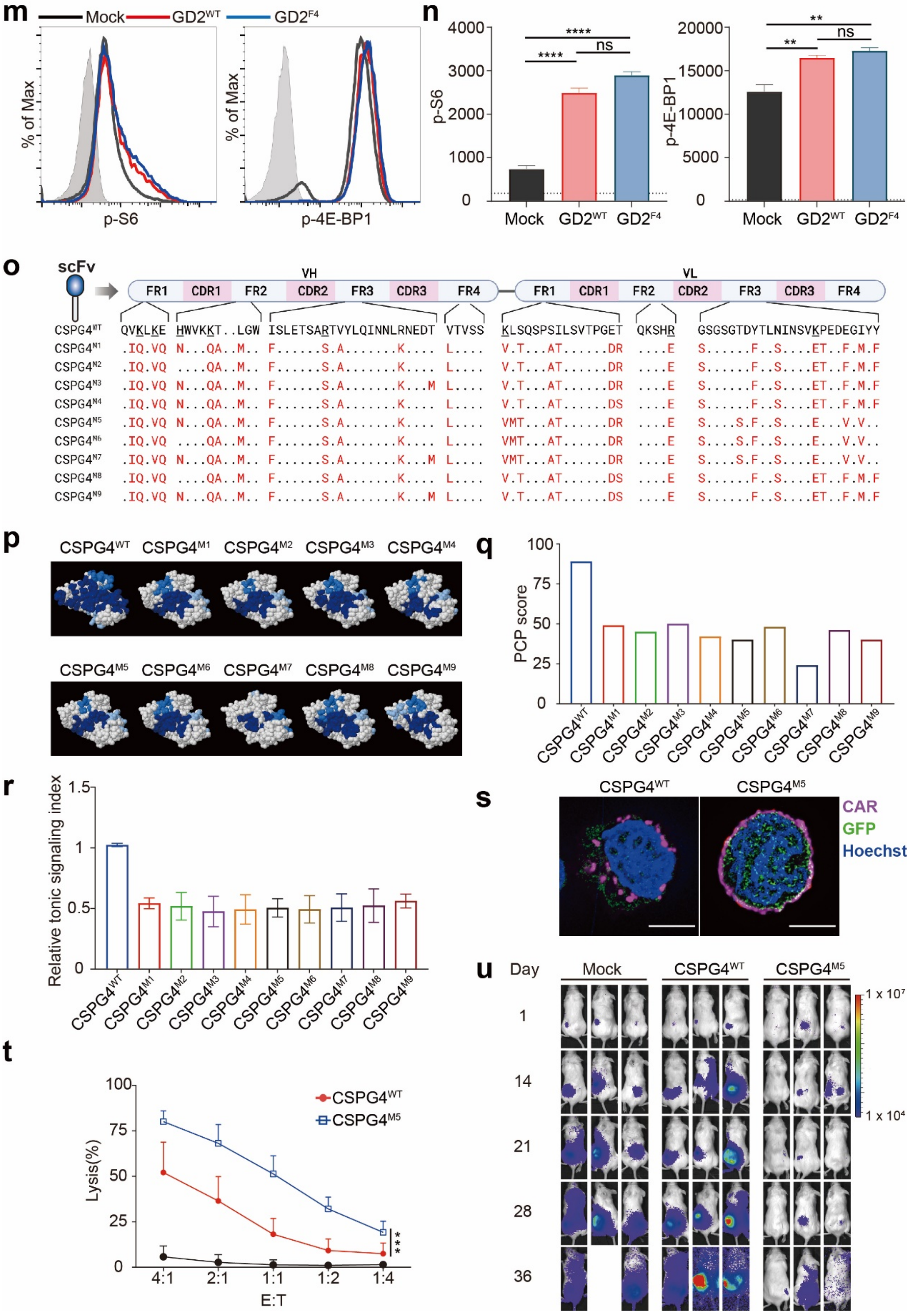
m
Mock
GD2^{WT}
GD2^{F4}
% of Max
p-S6
p-4E-BP1
n
p-S6
p-4E-BP1
****
**
ns
Mock
0
1000
2000
3000
4000
5000
10000
15000
20000
o
scFv
VH
VL
FR1
CDR1
FR2
CDR2
FR3
CDR3
FR4
CSPG4^{WT} QVKLKE HWVKKT..LGW ISLETSARTVYLQINNLRNEDT VTVSS KLSQSPSILSVTPGET QKSHR GSGSGTDYTLNINSVKPEDEGIYY
CSPG4^{M1} .IQ.VQ N...QA..M.. F......S.A.......K.... L.... V.T...AT......DR ....E S......F..S....ET..F.M.F
CSPG4^{M2} .IQ.VQ ....QA..M.. F......S.A.......K.... L.... V.T...AT......DR ....E S......F..S....ET..F.M.F
CSPG4^{M3} .IQ.VQ N...QA..M.. F......S.A.......K...M L.... V.T...AT......DR ....E S......F..S....ET..F.M.F
CSPG4^{M4} .IQ.VQ N...QA..M.. F......S.A.......K.... L.... V.T...AT......DS ....E S......F..S....ET..F.M.F
CSPG4^{M5} .IQ.VQ N...QA..M.. F......S.A.......K.... L.... VMT...AT......DR ....E S....S.F..S....E...V.V..
CSPG4^{M6} .IQ.VQ ....QA..M.. F......S.A.......K.... L.... VMT...AT......DR ....E S....S.F..S....E...V.V..
CSPG4^{M7} .IQ.VQ N...QA..M.. F......S.A.......K...M L.... VMT...AT......DR ....E S....S.F..S....E...V.V..
CSPG4^{M8} .IQ.VQ ....QA..M.. F......S.A.......K.... L.... V.T...AT......DS ....E S......F..S....ET..F.M.F
CSPG4^{M9} .IQ.VQ N...QA..M.. F......S.A.......K...M L.... V.T...AT......DS ....E S......F..S....ET..F.M.F
p
CSPG4^{WT} CSPG4^{M1} CSPG4^{M2} CSPG4^{M3} CSPG4^{M4}
CSPG4^{M5} CSPG4^{M6} CSPG4^{M7} CSPG4^{M8} CSPG4^{M9}
q
PCP score
0
25
50
75
100
r
Relative tonic signaling index
0
0.5
1
1.5
s
CSPG4^{WT}
CSPG4^{M5}
CAR
GFP
Hoechst
t
Lysis(%)
E:T
4:1
2:1
1:1
1:2
1:4
CSPG4^{WT}
CSPG4^{M5}
***
u
Day
Mock
CSPG4^{WT}
CSPG4^{M5}
1
14
21
28
36
1 x 10^{7}
1 x 10^{4}

Figure S4. Tuning down PCPs on GD2 or CSPG4 CAR surface mitigates T cell exhaustion and improves CAR-T efficacy.

(**a**) The sequences of initially optimized GD2.CAR variants.

(**b**) The top three largest PCPs on modified GD2.CAR scFv surface displayed by the BindUP web server tool. Dark blue: the first-largest PCP; medium blue: the second-largest PCP; light blue: the third-largest PCP.

(**c**) The calculation of PCP scores for the modified GD2.CAR scFvs.

(**d**) The calculation of relative tonic signaling indexes for the modified GD2.CAR scFvs.

(**e**) Electrostatics analysis of the optimized GD2.CAR scFv protein using APBS within UCSF Chimera. Blue, positively charged surface; red, negatively charged surface.

(**f**) The electrostatic potential fields of the optimized GD2.CAR scFv observed in the Swiss-PDBViewer software.

(**g-i**) Exhaustion markers including PD-1, LAG-3, and TIM-3 normalized by GFP expression in primary T cells expressing modified GD2.CAR were shown.

(**j**) Percentage of $TCF1^+$ progenitor exhausted cells in $Lag3^+TOX^+$ exhausted $GD2^{WT}$ and $GD2^{F4}$ CAR-T cells.

(**k-l**) Phosphorylation of ERK in $GD2^{WT}$ and $GD2^{F4}$ CAR-T cells measured by FACS.

(**m-n**) Phosphorylation of S6 and 4E-BP1 in $GD2^{WT}$ and $GD2^{F4}$ CAR-T cells assessed by FACS.

(**o**) The sequences of optimized CSPG4.CAR variants.

(**p**) The top three largest PCPs on optimized CSPG4.CAR scFv surface displayed by the BindUP web server tool. Dark blue: the first-largest PCP; medium blue: the second-largest PCP; light blue: the third-largest PCP.

(**q**) The calculation of PCP scores for the optimized CSPG4.CAR scFvs.

(**r**) The calculation of relative tonic signaling indexes for the optimized CSPG4.CAR scFvs.

(**s**) Imaging analysis of CAR clustering on $CSPG4^{WT}$ and $CSPG4^{M5}$ CAR-T cells. Pink: CAR; green: CAR-IRES EGFP; blue: Hoechst. Scale bars, 5μm.

(**t**) In vitro killing assay against $CSPG4^+$ nasopharyngeal cancer cell line CNE-2 of $CSPG4^{WT}$ and $CSPG4^{M5}$ CAR-T cells.

(**u**) Representative bioluminescence images of tumor burden after $CSPG4^{WT}$ and $CSPG4^{M5}$ CAR-T infusion over time.

Data are presented as means ± SEM; Comparisons were determined using unpaired student's t-tests (d, g-j, l, n) and two-way analysis of variance (t); * $P < 0.05$; ** $P < 0.01$; *** $P < 0.001$; **** $P < 0.0001$; ns not significant.
