# Supplementary material for: Tuning charge density of chimeric antigen receptor optimizes tonic signaling and CAR-T cell fitness
Source: Cell Res. 2023 Mar 8;33(5):341–54. doi: 10.1038/s41422-023-00789-0 (PMC10156745; doi:10.1038/s41422-023-00789-0)
Supplement: Supplementary file 5 — Fig. S5 [file 41422_2023_789_MOESM5_ESM.pdf]

Figure S5

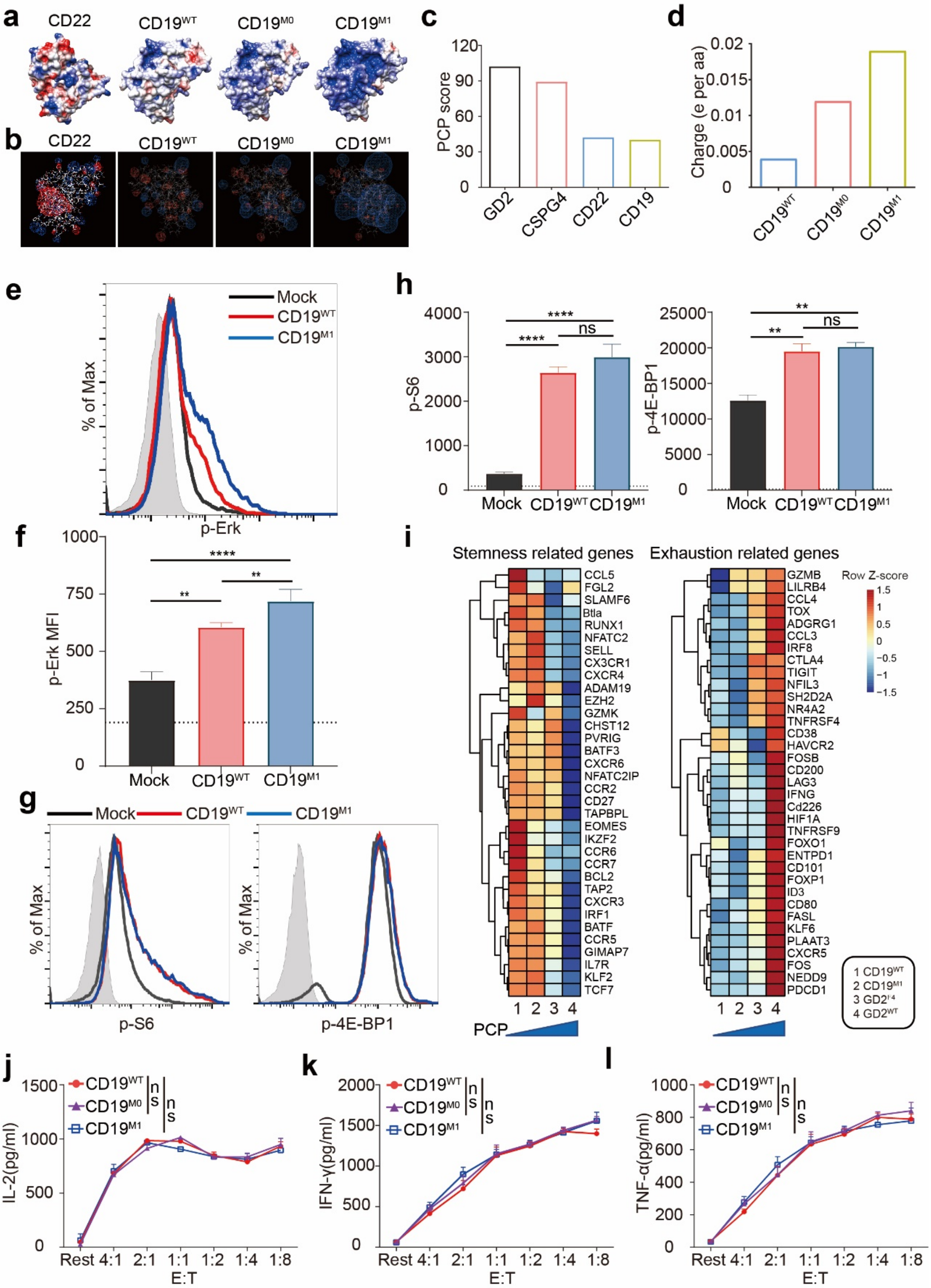
a
CD22
CD19WT
CD19M0
CD19M1
b
c
PCP score
GD2
CSPG4
CD22
CD19
d
Charge (e per aa)
e
Mock
% of Max
p-Erk
f
p-Erk MFI
g
p-S6
p-4E-BP1
h
ns
i
Stemness related genes
Exhaustion related genes
Row Z-score
PCP
1 CD19WT
2 CD19M1
3 GD2F4
4 GD2WT
j
IL-2(pg/ml)
k
IFN-γ(pg/ml)
l
TNF-α(pg/ml)
Rest
E:T

Figure S5. Tuning up PCPs on CD19 CAR surface enhances CAR-T cell fitness.

(**a**) Electrostatics analysis of CD22 and optimized CD19 CAR scFv constructs using APBS within UCSF Chimera. Blue, positively charged surface; red, negatively charged surface.

(**b**) The electrostatic potential fields of CD22 and optimized CD19 CAR scFv observed in the Swiss-PDBViewer software.

(**c**) The comparison of PCP scores between CD22 CAR and other CARs used in this study.

(**d**) Net amino acid charges of CD22 and optimized CD19 CAR scFvs at 0.15M Ionic strength and PH 7.5 calculated using the Protein–Sol webserver.

(**e-f**) Phosphorylation of ERK in $CD19^{WT}$ and $CD19^{M1}$ CAR-T cells measured by FACS.

(**g-h**) Phosphorylation of S6 and 4E-BP1 in $CD19^{WT}$ and $CD19^{M1}$ CAR-T cells detected by FACS.

(**i**) Expressions of stemness- and exhaustion-related genes in CAR-T cells.

(**j-l**) Cytokine secretion assay of optimized CD19 CAR-T cells after activation. Indicated CAR-T cells were harvested and co-incubated with K562-CD19 cells at the indicated E:T ratio. The levels of IL-2 and IFN-γ were determined by ELISA.

Data are presented as means ± SEM; Comparisons were determined using unpaired student’s t-tests (f, h) and two-way analysis of variance (j-l); ***P* < 0.01; **** *P* < 0.0001; ns not significant.
